# Continuous Glucose Monitoring and Hypoglycaemia Metrics With Once‐Weekly Basal Insulin Fc Versus Insulin Degludec: A Systematic Review and Meta‐Analysis

**DOI:** 10.1002/edm2.70067

**Published:** 2025-06-13

**Authors:** Saaed Abunada, Sheela Bai, Usha Kumari, F. N. U. Nancy, Areeba Khan, Nikeeta Bai, F. N. U. Shevani, Anusha Bai, Shah Dev, Noshad Zain ul Abiddin, F. N. U. Umer, Abdul Manan, Sadia Habib Bhutto, Salih Abdella Yusuf

**Affiliations:** ^1^ Liaquat University of Medical and Health Sciences Jamshoro, Karachi Pakistan; ^2^ United Medical and Dental College Karachi Pakistan; ^3^ Carroll High School Southlake Texas USA; ^4^ Khairpur Medical College Khairpur Pakistan; ^5^ Al‐Tibri Medical College Karachi Pakistan; ^6^ Liaquat National Hospital and Medical College Karachi Pakistan; ^7^ Ghulam Muhammad Mahar Medical College Sukkur Pakistan; ^8^ Kabir Medical College Peshawar Pakistan; ^9^ Jinnah Sindh Medical University Karachi Pakistan; ^10^ Shaheed Mohtarma Benazir Bhutto Medical College Lyari Karachi Pakistan; ^11^ Hawassa University Hawassa Ethiopia

**Keywords:** basal insulin Fc, continuous glucose monitoring, hypoglycaemia, insulin degludec

## Abstract

**Introduction:**

Once‐weekly basal insulin Fc (BIF) offers a promising alternative to daily basal insulin by reducing injection burden while maintaining glycaemic control. However, comprehensive comparisons with insulin degludec regarding continuous glucose monitoring (CGM) metrics and hypoglycaemia outcomes remain limited. This meta‐analysis evaluates these critical parameters.

**Methods:**

We conducted a systematic review and meta‐analysis of randomised controlled trials (RCTs) comparing once‐weekly BIF with once‐daily insulin degludec in type 1 and type 2 diabetes. Outcomes included CGM‐derived glycaemic variability, time in range, time above/below range and hypoglycaemia event rates. Data were pooled using random‐effects models, with heterogeneity assessed via *I* ^2^ statistics.

**Results:**

Five RCTs (*n* = 2427) were included. BIF demonstrated comparable glycaemic variability (within‐day CV: MD = 0.06, *p* = 0.90; between‐day CV: MD = ‐0.26, *p* = 0.30) and Time in range (MD = 0.56, *p* = 0.27) versus degludec. However, BIF increased time spent in the mild hypoglycaemia range (54–69 mg/dL) (MD = 0.30, *p* = 0.0004) and clinically significant hypoglycaemia event rates (rate ratio = 1.20, *p* < 0.00001). Severe hypoglycaemia event rates were higher with BIF (rate ratio = 3.34, *p* < 0.0001). Nocturnal hypoglycaemia and time above range (> 250 mg/dL) did not differ significantly.

**Conclusion:**

Once‐weekly BIF provides similar overall glycaemic control to insulin degludec but with increased time in mild hypoglycaemia and higher event rates of clinically significant and severe hypoglycaemia. These findings highlight the need for individualised dosing and monitoring when transitioning to weekly insulin regimens.

## Graphical abstract

This meta‐analysis highlights that once‐weekly basal insulin Fc provides comparable glycaemic control to daily insulin degludec but is associated with higher rates of clinically significant and severe hypoglycaemia, particularly in type 1 diabetes and insulin‐experienced patients.

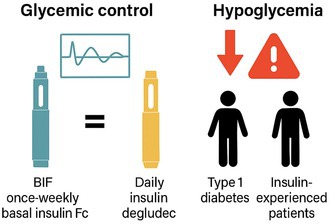

## 1 Introduction

Diabetes management is a dynamic and evolving field, particularly as the need for insulin therapy arises in both type 1 (T1D) and type 2 diabetes (T2D) [1, 2, 3]. In T1D, insulin is essential from the time of diagnosis, while in T2D, disease progression often necessitates the addition of basal insulin when glycaemic control cannot be maintained with non‐insulin glucose‐lowering agents alone [4, 5]. Despite clear clinical guidelines emphasising timely insulin initiation, there remains considerable hesitation among both patients and healthcare providers [6]. Concerns over injection burden, fear of hypoglycaemia, and the misconception that requiring insulin signifies treatment failure contribute to delays in initiation and suboptimal adherence, which can lead to poor glycaemic outcomes and increased risk of complications [7, 8].

Daily insulin injections present a challenge for individuals managing diabetes, particularly those requiring long‐term basal insulin therapy. Adherence to insulin regimens remains a significant barrier, with real‐world studies indicating that fewer than 30% of patients with T2D achieve the recommended HbA1c target of < 7% within a year of initiating basal insulin [9]. Similarly, only a minority of adults with T1D reach optimal glycaemic targets despite advances in insulin delivery methods and glucose monitoring technologies [10]. Reducing injection frequency has been associated with improved adherence and treatment persistence in other therapeutic areas, such as glucagon‐like peptide‐1 (GLP‐1) receptor agonists, where once‐weekly formulations have led to better glycaemic control compared to daily alternatives [11].

In response to these challenges, novel once‐weekly basal insulin formulations have been developed to reduce the burden of frequent injections while maintaining stable glycaemic control [12]. Basal insulin fc (BIF) (also known as Insulin efsitora alfa) is a fusion protein combining a single‐chain insulin variant with an immunoglobulin G Fc domain. The molecular design of BIF extends its half‐life to 17 days. This enables a flat pharmacokinetic profile and supports once‐weekly dosing [13]. Early clinical trials in both T1D and T2D have demonstrated that BIF provides effective glycaemic control comparable to daily basal insulin formulations, with similar rates of hypoglycaemia [14, 15, 16, 17, 18]. With its potential to improve adherence and simplify diabetes management, BIF represents a promising advancement.

A well‐conducted and comprehensive meta‐analysis has previously examined the efficacy and safety of BIF in comparison to insulin degludec in considerable detail [19]. However, important continuous glucose monitoring (CGM) outcomes—such as time above or below different glucose ranges—and hypoglycaemia outcomes, including nocturnal episodes and event rates, were not assessed. This meta‐analysis aims to address these gaps by providing a focused evaluation of these clinically relevant parameters.

## 2 Methods

### 2.1 Literature Search Strategy

This systematic review and meta‐analysis were conducted in accordance with the Preferred Reporting Items for Systematic Reviews and Meta‐Analyses (PRISMA) guidelines [20]. A comprehensive search was performed across multiple electronic databases, including PubMed, Google Scholar, Cochrane Library and ClinicalTrials.gov, covering studies published up to April 2025. The search strategy incorporated a combination of controlled vocabulary (e.g., MeSH terms) and free‐text keywords, including ‘type 1 diabetes’, ‘type 2 diabetes’, ‘basal insulin Fc’, ‘LY3209590’ and ‘once‐daily insulin degludec’. No language restrictions were applied. All retrieved articles were imported into EndNote, where duplicates were removed. A two‐stage screening process followed: first, the titles and abstracts were reviewed, and then the full texts of potentially eligible studies were assessed for eligibility. In addition, the reference lists of all included and relevant studies were thoroughly examined to identify any additional eligible studies that may have been missed during the initial search.

### 2.2 Eligibility Criteria

Randomised controlled trials (RCTs) were included if they met the following criteria:

1. *Population*: Patients with type 1 or type 2 diabetes, including insulin‐naive and insulin‐experienced individuals. Due to the limited number of available RCTs encompassing these subgroups, data were pooled to enable a more comprehensive synthesis of the evidence.
2. *Intervention*: Once‐weekly BIF.
3. *Comparison*: Once‐daily insulin degludec.
4. *Outcomes*: Reporting at least one of the following outcomes
  - *CGM outcomes*: Outcomes included within‐day and between‐day glycaemic variability (CV, %), time in range (70–180 mg/dL), time below range (< 54 mg/dL, 54–69 mg/dL) and time above range (180–250 mg/dL, > 250 mg/dL).
  - *Hypoglycaemic outcomes*: Assessed outcomes included hypoglycaemia alert rate, clinically significant hypoglycaemia rate, severe hypoglycaemia rate, hypoglycaemia alert event rate, clinically significant hypoglycaemia event rate, severe hypoglycaemia event rate, nocturnal hypoglycaemia alert rate, nocturnal clinically significant hypoglycaemia rate, nocturnal hypoglycaemia alert event rate and nocturnal clinically significant hypoglycaemia event rate. To aid interpretation, it is important to note that ‘rate’ refers to the proportion of participants experiencing at least one episode, whereas ‘event rate’ accounts for the total number of episodes over time, including recurrent events.

Exclusion criteria included non‐randomised trials, observational studies, case reports and studies lacking necessary outcome data.

### 2.3 Data Extraction

Two reviewers independently performed data extraction from the eligible trials. The extracted information was systematically organised into a standardised data collection table

- *Study characteristics*: Included trial identifiers (e.g., NCT number), study phase and publication year, diabetes classification, prior insulin use, type of insulin administered and duration of follow‐up.
- *Participant demographics*: Encompassed baseline characteristics such as age, sex, weight, body mass index (BMI), HbA1c (%), fasting serum glucose (mg/dL), duration of diabetes and fasting blood glucose (FBG) target range (mg/dL).
- *Outcomes*: Included CGM and hypoglycaemic outcomes

Any discrepancies encountered during data extraction were resolved through discussion and mutual agreement; if consensus could not be reached, a third reviewer was consulted for adjudication.

### 2.4 Risk of Bias Assessment

The methodological quality of included RCTs was evaluated using the Cochrane Risk of Bias tool version 2 (RoB 2), which assesses five domains: randomisation process, deviations from intended interventions, missing outcome data, outcome measurement and selective reporting [21]. Each domain was judged as having low, moderate, or high risk of bias. The overall risk of bias for each study was determined based on the most critical limitation identified across all domains. Two reviewers independently conducted the quality assessments, with disagreements resolved through consensus.

### 2.5 Statistical Analysis

Meta‐analysis was conducted using RevMan 5.4. For continuous outcomes, mean differences (MD) with 95% confidence intervals (CI) were calculated using the inverse variance method, while dichotomous outcomes were pooled using risk ratios (RR); event rate outcomes were analysed using rate ratios. A random‐effects model was applied throughout to account for potential clinical and methodological heterogeneity. Statistical heterogeneity was assessed using the *I* ^2^ statistic, with thresholds of < 50% indicating low, 50%–75% moderate and > 75% high heterogeneity. Sensitivity analyses were performed by sequentially excluding studies to evaluate their influence on pooled estimates, particularly for outcomes with substantial heterogeneity (*I* ^2^ > 75%) [22]. Pre‐specified subgroup analyses were conducted based on diabetes type (type 1 vs. type 2), insulin treatment status (naive vs. previously treated) and follow‐up duration (26, 32 and 52 weeks). Forest plots were used for visual representation of results. Assessment of publication bias was not performed, as fewer than 10 studies were available per outcome—below the commonly accepted threshold for reliable interpretation of funnel plots or Egger's test. A *p*‐value of < 0.05 was considered statistically significant for all analyses.

## 3 Results

### 3.1 Identification and Selection of Studies

**FIGURE 1:**
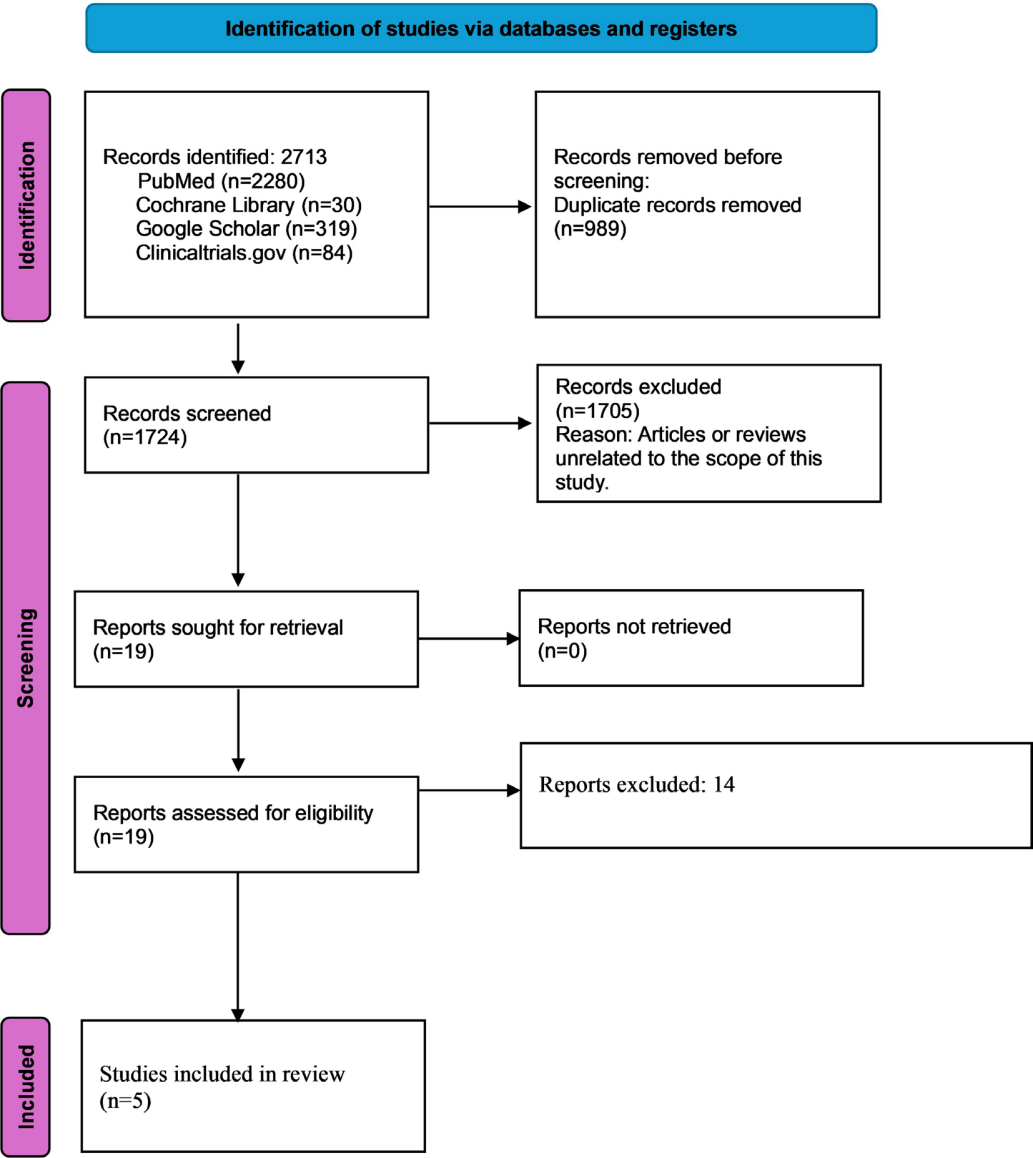
PRISMA flow diagram depicting the study selection procedure.

A total of 2713 records were identified through database searches, including PubMed, Cochrane Library, Google Scholar and ClinicalTrials.gov. After the removal of 989 records, 1724 were assessed. Of these, 1705 were excluded following title and abstract screening. Nineteen full texts were reviewed, with 14 subsequently excluded. Ultimately, five studies were included in the synthesis [14, 15, 16, 17, 18]. The study selection process is illustrated in Figure 1.

### 3.2 Study and Patient Characteristics

This meta‐analysis included five RCTs [14, 15, 16, 17, 18] comparing once‐weekly BIF with once‐daily insulin degludec, published between 2023 and 2024, comprising both Phase II and Phase III studies (Table 1). Across the trials, a total of 2427 participants with either type 1 or type 2 diabetes were enrolled. Two studies included insulin‐naive individuals [15, 17], while three included previously insulin‐treated participants [14, 16, 18]. Mean participant age ranged from 43.6 to 60.8 years, with higher values observed in T2D cohorts. Across studies, mean body weight and BMI ranged from 74.8 to 90.6 kg and 25.9 to 32.4 kg/m^2^, respectively. Baseline HbA1c levels were generally well‐matched between arms, ranging from 7.5% to 8.23%. Fasting serum glucose levels ranged from 141.7 to 170.2 mg/dL, with target fasting glucose values set between 80 and 120 mg/dL. The duration of diabetes varied from 9.7 to 22.3 years, and follow‐up ranged from 26 to 52 weeks (Table 1).

**TABLE 1:** Study and baseline characteristics.

Study	Phase and year	Diabetes type	Previously insulin treated	Insulin type	Participants (*N*)	Sex (*N*)	Age (years)	Weight (kg)	HbA1c (%)	Fasting serum glucose (mg/dL)	FBG target range (mg/dL)	BMI (kg/m^2^)	Follow up
						(M/F)	(mean ± SD)	(mean ± SD)	(mean ± SD)	(mean ± SD)		(mean ± SD)	
Bergenstal, R. M. et al.	Phase III 2024	T1D	Yes	Insulin efsitora	343	193/150	44·4 ± 14·2	76·2 ± 15·6	7·88 ± 0·75	157.2 ± 68.15	80–120	26·5 ± 4·0	52 weeks
				Insulin degludec	349	191/158	43·6 ± 14·0	74·8 ± 15·9	7·94 ± 0·72	164·0 ± 71.11	80–120	25·9 ± 4·1	
Bue‐Valleskey, J. M. et al.	Phase II 2023	T2D	No	Insulin efsitora	143	76/67	57.3 ± 9.7	88.4 ± 19.8	8.1 ± 0.8	170.2 ± 42.0	80–100	32.3 ± 5.4	26 weeks
				Insulin degludec	135	76/59	59.4 ± 9.1	90.6 ± 19.6	8.0 ± 0.8	160.7 ± 36.7	80–100	31.6 ± 5.5	
Frias, J. et al.	Phase II 2023	T2D	Yes	Insulin efsitora	132	62/70	59·6 ± 11·3	88·1 ± 18·9	8·0 ± 0·9	141·7 ± 47·5	≤ 120	32·4 ± 5·8	32 weeks
				Insulin degludec	132	67/65	60·8 ± 10·0	87·1 ± 20·7	8·1 ± 0·9	144·5 ± 51·0	≤ 100	31·8 ± 5·7	
Kazda, C. M. et al.	Phase II 2023	T1D	Yes	Insulin efsitora	139	86/53	45.5 ± 15.3	81.3 ± 16.0	7.5 ± 0.8	165.4 ± 67.9	80–100	27.5 ± 4.0	26 weeks
				Insulin degludec	126	78/48	47.4 ± 13.7	82.0 ± 15.1	7.5 ± 0.9	159.3 ± 67.1	80–100	27.2 ± 4.1	
Wysham, C. et al.	Phase III 2024	T2D	No	Insulin efsitora	466	281/185	57.6 ± 10.6	86.83 ± 20.53	8.21 ± 0.96	162.32 ± 45.79	80–120	30.44 ± 5.85	52 weeks
				Insulin degludec	462	265/197	57.3 ± 11.0	86.12 ± 18.93	8.23 ± 0.96	165.13 ± 48.78	80–120	30.72 ± 5.90	

Abbreviations: %, percentage; BMI, body mass index; dL, deciliter; F, female; FBG, fasting blood glucose; kg, kilogram; M, male; m^2^, square meter; mg, milligram; *N*, number; SD, Standard deviations; T1D, type 1 diabetes; T2D, type 2 diabetes.

### 3.3 Bias Assessment

**FIGURE 2:**
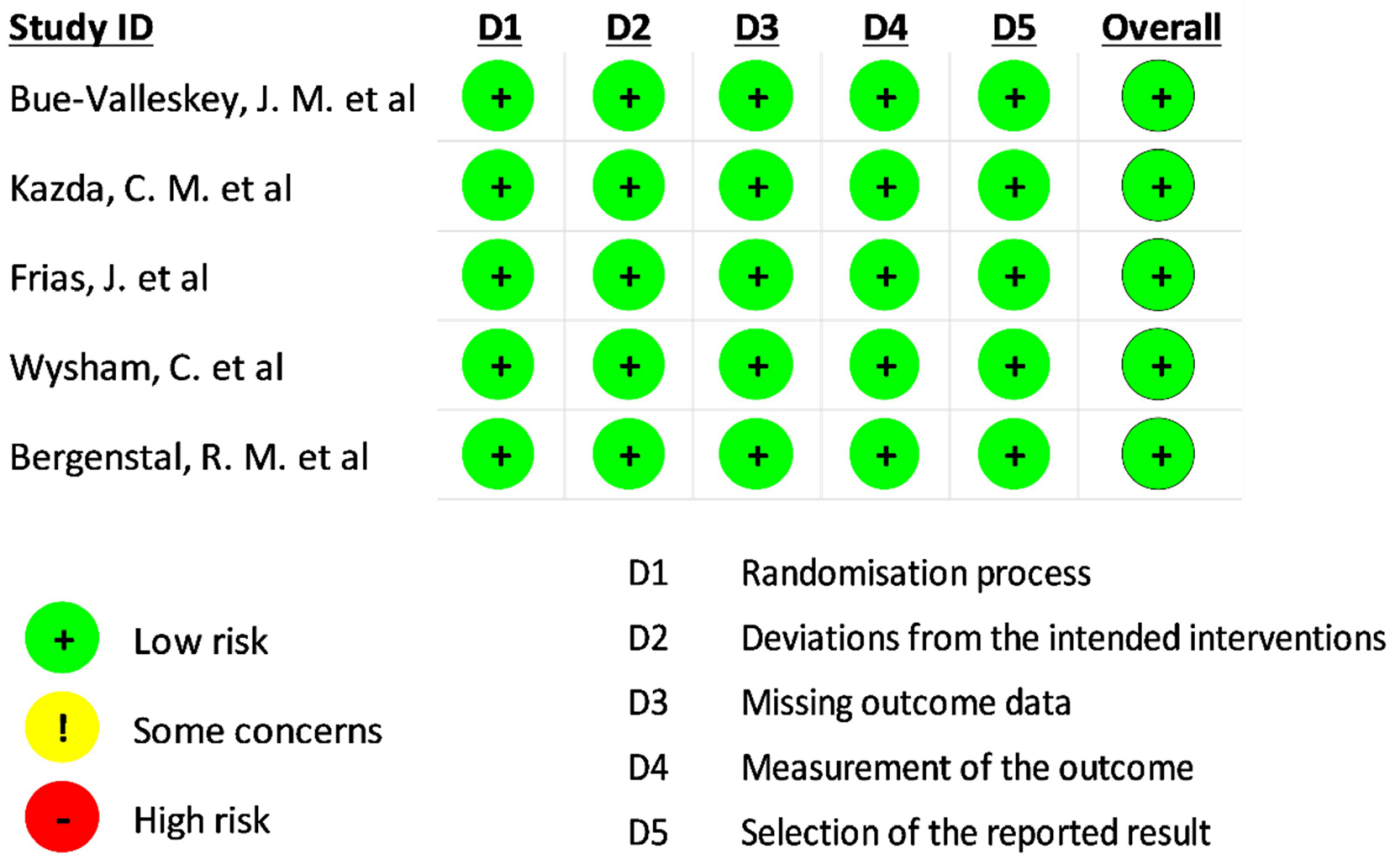
Assessment of risk of bias for the included randomised controlled trials (RCTs).

All five included RCTs demonstrated a low risk of bias across all five domains, as illustrated in Figure 2. Although the trials were open‐label due to the differing insulin administration schedules (once‐weekly BIF vs. once‐daily degludec), the use of objective outcome measures mitigated the potential for performance or detection bias. Consequently, all studies were rated as low risk of bias overall.

#### 3.3.1 Glycaemic Variability Within‐Day (CV, %)

**FIGURE 3:**
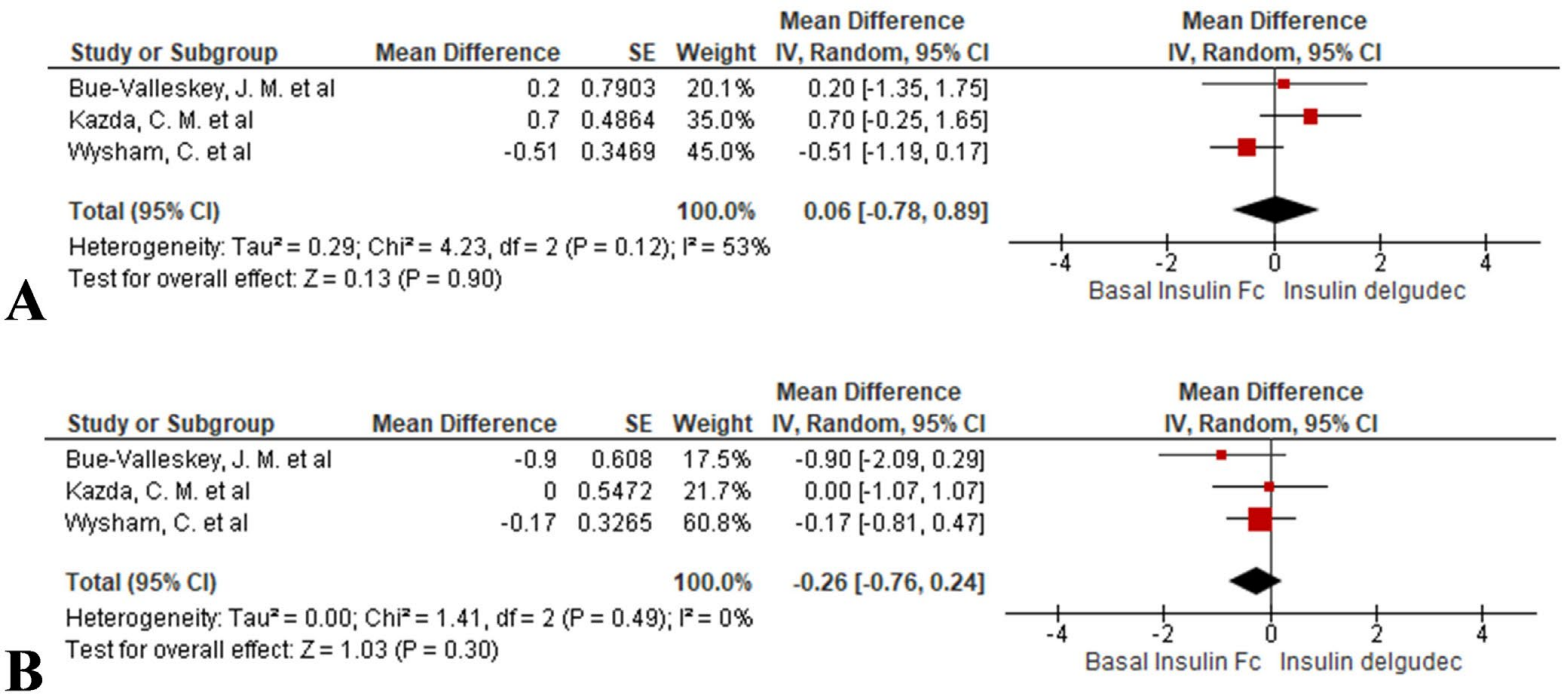
Forest plots comparing glycaemic variability between BIF and insulin degludec: (A) within‐day (CV, %) and (B) between‐day (CV, %).

BIF showed no significant difference from insulin degludec (MD = 0.06, 95% CI: −0.78 to 0.89; *I* ^2^ = 53%, *p* = 0.90; Figure 3A).

#### 3.3.2 Glycaemic Variability Between‐Day (CV, %)

The comparison between BIF and insulin degludec revealed no meaningful difference (MD = −0.26, 95% CI: −0.76 to 0.24; *I* ^2^ = 0%, *p* = 0.30; Figure 3B).

#### 3.3.3 Time in Range (%)

**FIGURE 4:**
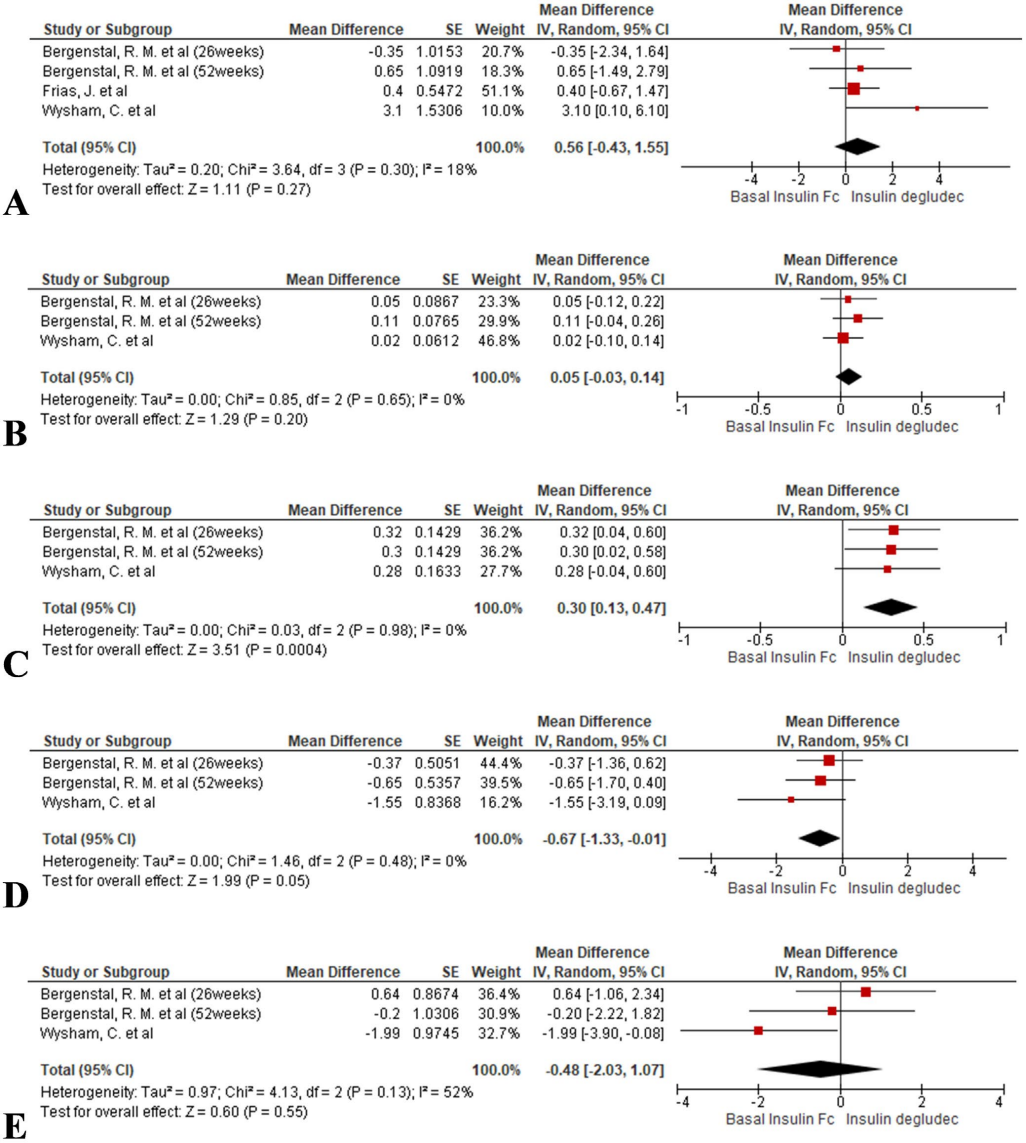
Forest plots comparing CGM‐derived time‐in‐range metrics between BIF and insulin degludec: (A) Time in range (70–180 mg/dL), (B) < 54 mg/dL, (C) 54–69 mg/dL, (D) 180–250 mg/dL and (E) > 250 mg/dL.

Time in range remained similar across groups (MD = 0.56, 95% CI: −0.43 to 1.55; *I* ^2^ = 18%, *p* = 0.27; Figure 4A).

#### 3.3.4 Time Below Range (< 54 mg/dL, %)

BIF did not significantly alter time spent below 54 mg/dL compared to insulin degludec (MD = 0.05, 95% CI: −0.03 to 0.14; *I* ^2^ = 0%, *p* = 0.20; Figure 4B).

#### 3.3.5 Time Below Range (54–69 mg/dL, %)

BIF was associated with a modest increase in time below this range (MD = 0.30, 95% CI: 0.13 to 0.47; *I* ^2^ = 0%, *p* = 0.0004; Figure 4C).

#### 3.3.6 Time Above Range (180–250 mg/dL, %)

A borderline reduction in time above range was noted with BIF (MD = −0.67, 95% CI: −1.33 to −0.01; *I* ^2^ = 0%, *p* = 0.05; Figure 4D).

#### 3.3.7 Time Above Range (> 250 mg/dL, %)

No appreciable difference was seen between BIF and insulin degludec in time spent above 250 mg/dL (MD = −0.48, 95% CI: −2.03 to 1.07; *I* ^2^ = 52%, *p* = 0.55; Figure 4E).

### 3.4 Hypoglycaemic Outcomes

#### 3.4.1 Hypoglycaemia Alert

**FIGURE 5:**
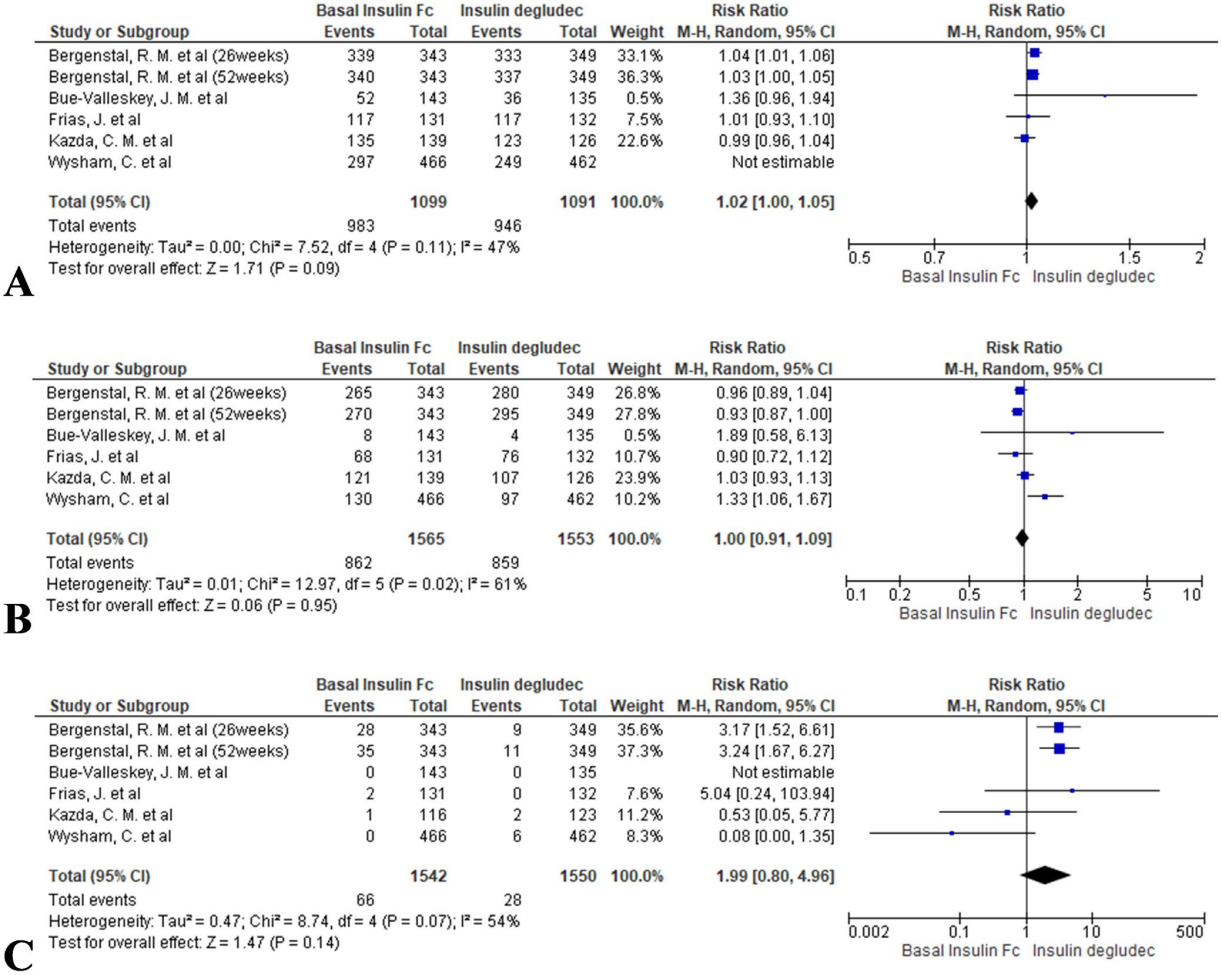
Forest plots comparing hypoglycaemia rates between BIF and insulin degludec: (A) hypoglycaemia alert, (B) clinically significant hypoglycaemia and (C) severe hypoglycaemia.

The RR for hypoglycaemia alert was 1.02 (95% CI: 1.00 to 1.05; *I* ^2^ = 47%, *p* = 0.09; Figure 5A), indicating no statistically significant difference.

#### 3.4.2 Clinically Significant Hypoglycaemia

Rates were nearly identical between groups (RR = 1.00, 95% CI: 0.91 to 1.09; *I* ^2^ = 61%, *p* = 0.95; Figure 5B).

#### 3.4.3 Severe Hypoglycaemia

BIF showed a non‐significant trend toward an increased risk of severe hypoglycaemia (RR = 1.99, 95% CI: 0.80 to 4.96; *I* ^2^ = 54%, *p* = 0.14; Figure 5C).

#### 3.4.4 Hypoglycaemia Alert Event Rate

**FIGURE 6:**
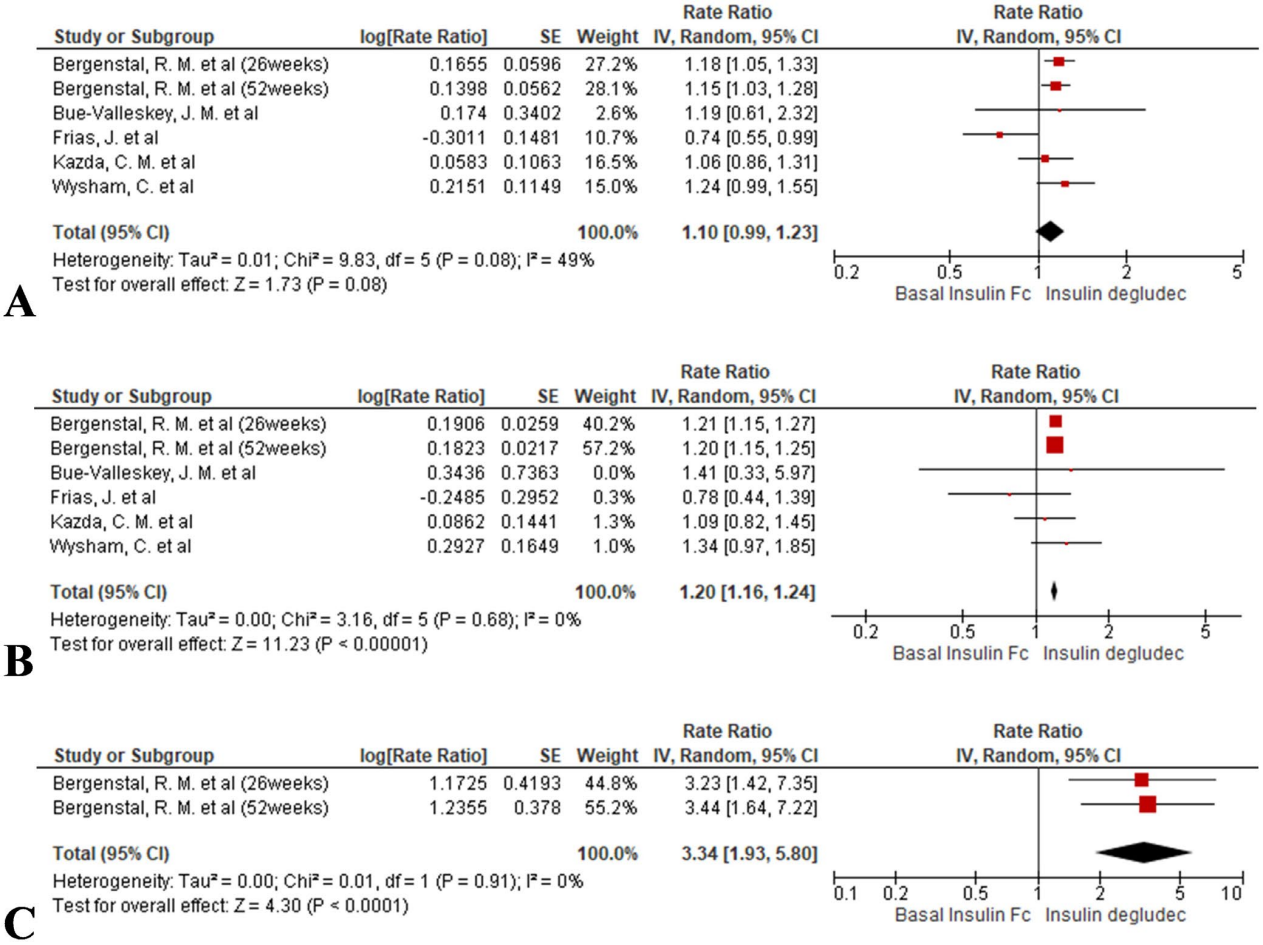
Forest plots comparing hypoglycaemia event rates between BIF and insulin degludec: (A) hypoglycaemia alert events, (B) clinically significant hypoglycaemia events and (C) severe hypoglycaemia events.

Event rates were comparable between BIF and insulin degludec (rate ratio = 1.10, 95% CI: 0.99 to 1.23; *I* ^2^ = 49%, *p* = 0.08; Figure 6A).

#### 3.4.5 Clinically Significant Hypoglycaemia Event Rate

A significantly higher event rate was observed with BIF (rate ratio = 1.20, 95% CI: 1.16 to 1.24; *I* ^2^ = 0%, *p* < 0.00001; Figure 6B).

#### 3.4.6 Severe Hypoglycaemia Event Rate

BIF resulted in a markedly higher rate of severe hypoglycaemia events (rate ratio = 3.34, 95% CI: 1.93 to 5.80; *I* ^2^ = 0%, *p* < 0.0001; Figure 6C).

#### 3.4.7 Nocturnal Hypoglycaemia Alert

**FIGURE 7:**
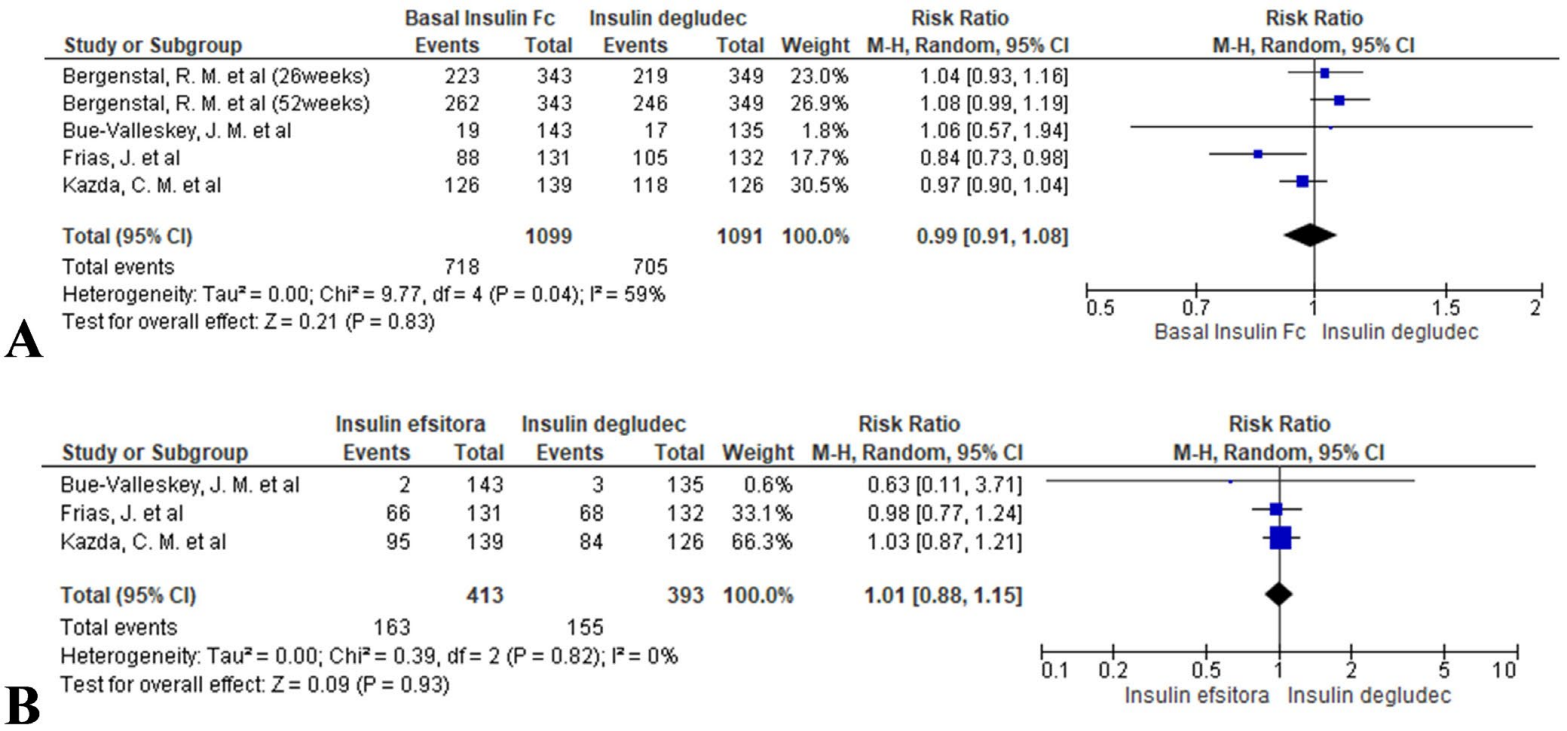
Forest plots comparing nocturnal hypoglycaemia rates between BIF and insulin degludec: (A) hypoglycaemia alert rates and (B) clinically significant hypoglycaemia rates.

The two groups demonstrated similar rates (RR = 0.99, 95% CI: 0.91 to 1.08; *I* ^2^ = 59%, *p* = 0.83; Figure 7A).

#### 3.4.8 Nocturnal Clinically Significant Hypoglycaemia

No meaningful difference was detected (RR = 1.01, 95% CI: 0.88 to 1.15; *I* ^2^ = 0%, *p* = 0.93; Figure 7B).

#### 3.4.9 Nocturnal Hypoglycaemia Alert Event Rate

**FIGURE 8:**
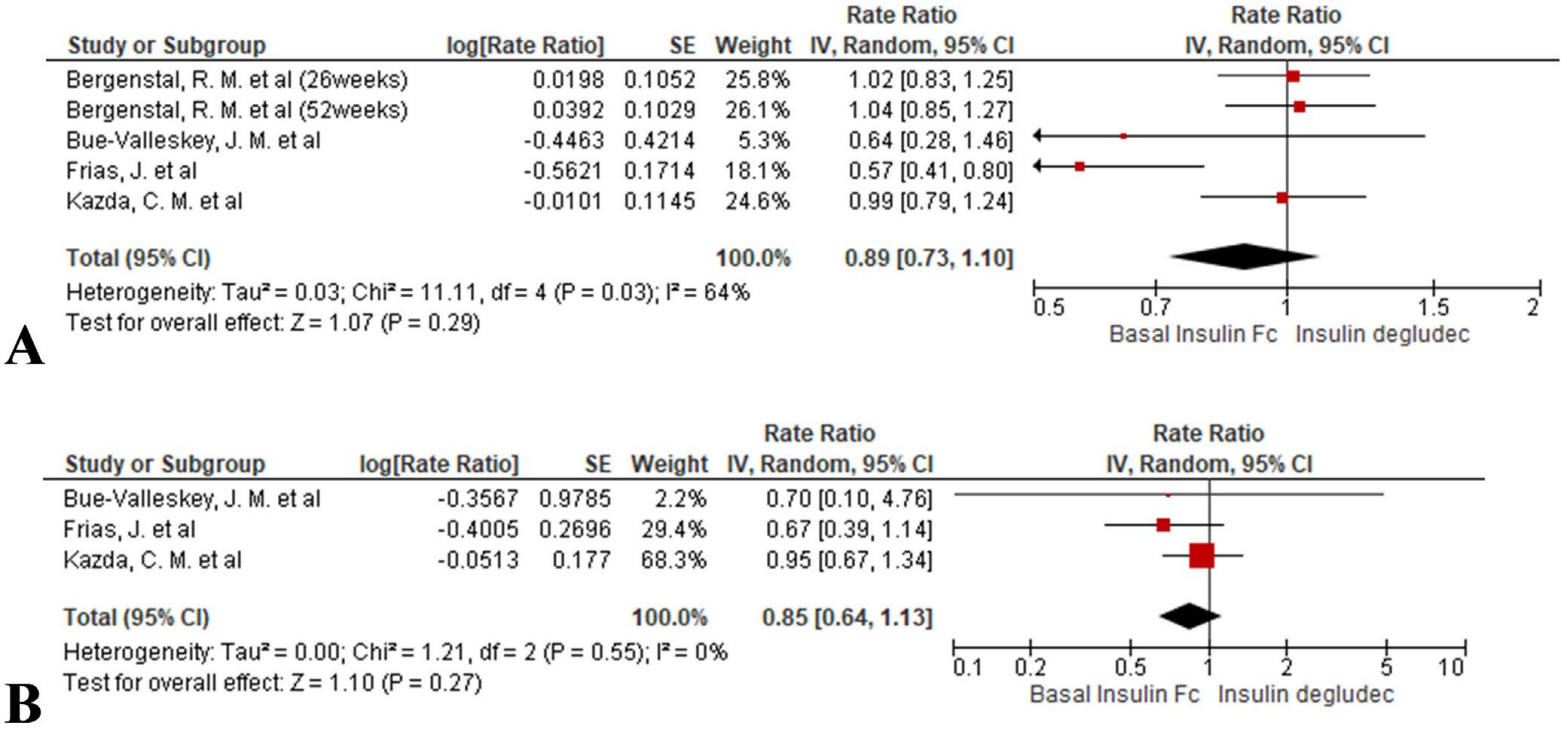
Forest plots comparing nocturnal hypoglycaemia event rates between BIF and insulin degludec: (A) hypoglycaemia alert event rates and (B) clinically significant hypoglycaemia event rates.

Event frequency did not differ significantly between BIF and insulin degludec (rate ratio = 0.89, 95% CI: 0.73 to 1.10; *I* ^2^ = 64%, *p* = 0.29; Figure 8A).

#### 3.4.10 Nocturnal Clinically Significant Hypoglycaemia Event Rate

The event rate of nocturnal clinically significant episodes remained similar across both groups (rate ratio = 0.85, 95% CI: 0.64 to 1.13; *I* ^2^ = 0%, *p* = 0.27; Figure 8B).

### 3.5 Subgroup Analysis

#### 3.5.1 By Diabetes Type (Table 2)

**TABLE 2:** Subgroup analysis based on diabetes type (type 1 diabetes or type 2 diabetes).

Outcome	Diabetes type	Mean difference (MD) or risk ratio (RR) or odds ratio (OR) or rate ratio	95% CI	*p*	*I* ^2^	Figure reference
Glycaemic variability within‐day (CV, %)	T1D	MD: 0.70	−0.25, 1.65	0.15	NA	Figure S1
	T2D	MD: −0.40	−1.02, 0.23	0.21	0%	
Glycaemic variability between‐day (CV, %)	T1D	MD: 0.00	−1.07, 1.07	1	NA	Figure S2
	T2D	MD: −0.35	−0.98, 0.27	0.26	11%	
Time in range (70–180 mg/dL, %)	T1D	MD: 0.11	−1.34, 1.57	0.88	0%	Figure S3
	T2D	MD: 1.37	−1.17, 3.91	0.29	64%	
Time below range (< 54 mg/dL, %)	T1D	MD: 0.08	−0.03, 0.20	0.14	0%	Figure S4
	T2D	MD: 0.02	−0.10, 0.14	0.74	NA	
Time below range (54–69 mg/dL, %)	T1D	MD: 0.31	0.11, 0.51	0.002	0%	Figure S5
	T2D	MD: 0.28	−0.04, 0.60	0.09	NA	
Time above range (180–250 mg/dL, %)	T1D	MD: −0.50	−1.22, 0.22	0.17	0%	Figure S6
	T2D	MD: −1.55	−3.19, 0.09	0.06	NA	
Time above range (> 250 mg/dL, %)	T1D	MD: 0.29	−1.01, 1.59	0.66	0%	Figure S7
	T2D	MD: −1.99	−3.90, −0.08	0.04	NA	
Hypoglycaemia alert	T1D	RR: 1.02	1.00, 1.04	0.02	30%	Figure S8
	T2D	RR: 1.13	0.94, 1.36	0.18	81%	
Clinically significant hypoglycaemia	T1D	RR: 0.96	0.92, 1.02	0.17	18%	Figure S9
	T2D	RR: 1.14	0.79, 1.65	0.47	71%	
Severe hypoglycaemia	T1D	RR: 2.96	1.79, 4.89	< 0.0001	5%	Figure S10
	T2D	RR: 0.60	0.01, 37.80	0.81	75%	
Hypoglycaemia alert event rate	T1D	Rate ratio: 1.15	1.07, 1.24	0.0002	0%	Figure S11
	T2D	Rate ratio: 1.01	0.68, 1.50	0.96	74%	
Clinically significant hypoglycaemia event rate	T1D	Rate ratio: 1.20	1.16, 1.24	< 0.00001	0%	Figure S12
	T2D	Rate ratio: 1.14	0.78, 1.65	0.5	24%	
Severe hypoglycaemia event rate	T1D	Rate ratio: 3.34	1.93, 5.80	< 0.0001	0%	Figure S13
	T2D	—	—	—	—	
Nocturnal hypoglycaemia alert	T1D	RR: 1.02	0.94, 1.11	0.57	60%	Figure S14
	T2D	RR: 0.85	0.74, 0.99	0.03	0%	
Nocturnal clinically significant hypoglycaemia	T1D	RR: 1.03	0.87, 1.21	0.77	NA	Figure S15
	T2D	RR: 0.97	0.77, 1.23	0.8	0%	
Nocturnal hypoglycaemia alert event rate	T1D	Rate ratio: 1.02	0.90, 1.15	0.77	0%	Figure S16
	T2D	Rate ratio: 0.58	0.42, 0.79	0.0006	0%	
Nocturnal clinically significant hypoglycaemia event rate	T1D	Rate ratio: 0.95	0.67, 1.34	0.77	NA	Figure S17
	T2D	Rate ratio: 0.67	0.40, 1.12	0.13	0%	

##### 3.5.1.1 T1D

Time below range (54–69 mg/dL) was significantly higher. Alert hypoglycaemia events and rates were significantly elevated. Clinically significant hypoglycaemia event rate was increased. Severe hypoglycaemia and its event rate were markedly elevated.

##### 3.5.1.2 T2D

Time above range (> 250 mg/dL) was significantly reduced. Alert nocturnal hypoglycaemia was significantly lower. Event rate for alert nocturnal hypoglycaemia was significantly reduced.

#### 3.5.2 By Insulin Status (Table 3)

**TABLE 3:** Subgroup analysis based on insulin status (insulin‐naive or previously insulin‐treated).

Outcome	Insulin status	Mean difference (MD) or risk ratio (RR) or odds ratio (OR) or rate ratio	95% CI	*p*	*I* ^2^	Figure reference
Glycaemic variability within‐day (CV, %)	Insulin‐naive	MD: −0.40	−1.02, 0.23	0.21	0%	Figure S18
	Previously insulin‐treated	MD: 0.70	−0.25, 1.65	0.15	NA	
Glycaemic variability between‐day (CV, %)	Insulin‐naïve	MD: −0.35	−0.98, 0.27	0.26	11%	Figure S19
	Previously insulin‐treated	MD: 0.00	−1.07, 1.07	1	NA	
Time in range (70–180 mg/dL, %)	Insulin‐naïve	MD: 3.10	0.10, 6.10	0.04	NA	Figure S20
	Previously insulin‐treated	MD: 0.30	−0.56, 1.16	0.5	0%	
Time below range (< 54 mg/dL, %)	Insulin‐naïve	MD: 0.02	−0.10, 0.14	0.74	NA	Figure S21
	Previously insulin‐treated	MD: 0.08	−0.03, 0.20	0.14	0%	
Time below range (54–69 mg/dL, %)	Insulin‐naïve	MD: 0.28	−0.04, 0.60	0.09	NA	Figure S22
	Previously insulin‐treated	MD: 0.31	0.11, 0.51	0.002	0%	
Time above range (180–250 mg/dL, %)	Insulin‐naïve	MD: −1.55	−3.19, 0.09	0.06	NA	Figure S23
	Previously insulin‐treated	MD: −0.50	−1.22, 0.22	0.17	0%	
Time above range (> 250 mg/dL, %)	Insulin‐naïve	MD: −1.99	−3.90, −0.08	0.04	NA	Figure S24
	Previously insulin‐treated	MD: 0.29	−1.01, 1.59	0.66	0%	
Hypoglycaemia alert	Insulin‐naïve	RR: 1.20	1.08, 1.33	0.0007	0%	Figure S25
	Previously insulin‐treated	RR: 1.02	1.01, 1.04	0.002	1%	
Clinically significant hypoglycaemia	Insulin‐naïve	RR: 1.35	1.07, 1.69	0.01	0%	Figure S26
	Previously insulin‐treated	RR: 0.96	0.92, 1.00	0.08	0%	
Severe hypoglycaemia	Insulin‐naïve	RR: 0.08	0.00, 1.35	0.08	NA	Figure S27
	Previously insulin‐treated	RR: 3.02	1.88, 4.86	< 0.00001	0%	
Hypoglycaemia alert event rate	Insulin‐naïve	Rate ratio: 1.23	1.00, 1.53	0.05	0%	Figure S28
	Previously insulin‐treated	Rate ratio: 1.07	0.93, 1.23	0.37	67%	
Clinically significant hypoglycaemia event rate	Insulin‐naïve	Rate ratio: 1.34	0.98, 1.84	0.07	0%	Figure S29
	Previously insulin‐treated	Rate ratio: 1.20	1.16, 1.24	< 0.00001	0%	
Severe hypoglycaemia event rate	Insulin‐naïve	—	—	—	—	Figure S30
	Previously insulin‐treated	Rate ratio: 3.34	1.93, 5.80	< 0.0001	0%	
Nocturnal hypoglycaemia alert	Insulin‐naïve	RR: 1.06	0.57, 1.94	0.86	NA	Figure S31
	Previously insulin‐treated	RR: 0.99	0.90, 1.08	0.81	69%	
Nocturnal clinically significant hypoglycaemia	Insulin‐naïve	RR: 0.63	0.11, 3.71	0.61	NA	Figure S32
	Previously insulin‐treated	RR: 1.01	0.88, 1.16	0.9	0%	
Nocturnal hypoglycaemia alert event rate	Insulin‐naïve	Rate ratio: 0.64	0.28, 1.46	0.29	NA	Figure S33
	Previously insulin‐treated	Rate ratio: 0.91	0.73, 1.13	0.39	71%	
Nocturnal clinically significant hypoglycaemia event rate	Insulin‐naïve	Rate ratio: 0.70	0.10, 4.76	0.72	NA	Figure S34
	Previously insulin‐treated	Rate ratio: 0.85	0.61, 1.17	0.31	15%	

##### 3.5.2.1 Insulin‐Naive

Time in range was significantly improved. Time above range (> 250 mg/dL) was significantly reduced. Risk of alert and clinically significant hypoglycaemia was elevated.

##### 3.5.2.2 Previously Insulin‐Treated

Time below range (54–69 mg/dL) was significantly higher. Risk of alert and severe hypoglycaemia was elevated. Event rate of clinically significant and severe hypoglycaemia was significantly higher.

#### 3.5.3 By Follow‐Up Duration (Table 4)

**TABLE 4:** Subgroup analysis based on the follow‐up duration (26, 32 and 52 weeks).

Outcome	Follow‐up duration	Mean difference (MD) or risk ratio (RR) or odds ratio (OR) or rate ratio	95% CI	*p*	*I* ^2^	Figure reference
Glycaemic variability within‐day (CV, %)	26 weeks	MD: 0.56	−0.25, 1.37	0.17	0%	Figure S35
	32 weeks	—	—	—	—	
	52 weeks	MD: −0.51	−1.19, 0.17	0.14	NA	
Glycaemic variability between‐day (CV, %)	26 weeks	MD: −0.41	−1.29, 0.47	0.36	17%	Figure S36
	32 weeks	—	—	—	—	
	52 weeks	MD: −0.17	−0.81, 0.47	0.6	NA	
Time in range (70–180 mg/dL, %)	26 weeks	MD: −0.35	−2.34, 1.64	0.73	NA	Figure S37
	32 weeks	MD: 0.40	−0.67, 1.47	0.46	NA	
	52 weeks	MD: 1.64	−0.72, 4.00	0.17	41%	
Time below range (< 54 mg/dL, %)	26 weeks	MD: 0.05	−0.12, 0.22	0.56	NA	Figure S38
	32 weeks	—	—	—	—	
	52 weeks	MD: 0.06	−0.04, 0.15	0.25	0%	
Time below range (54–69 mg/dL, %)	26 weeks	MD: 0.32	0.04, 0.60	0.03	NA	Figure S39
	32 weeks	—	—	—	—	
	52 weeks	MD: 0.29	0.08, 0.50	0.007	0%	
Time above range (180–250 mg/dL, %)	26 weeks	MD: −0.37	−1.36, 0.62	0.46	NA	Figure S40
	32 weeks	—	—	—	—	
	52 weeks	MD: −0.91	−1.80, −0.03	0.04	0%	
Time above range (> 250 mg/dL, %)	26 weeks	MD: 0.64	−1.06, 2.34	0.46	NA	Figure S41
	32 weeks	—	—	—	—	
	52 weeks	MD: −1.13	−2.88, 0.63	0.21	37%	
Hypoglycaemia alert	26 weeks	RR: 1.03	0.96, 1.10	0.45	80%	Figure S42
	32 weeks	RR: 1.01	0.93, 1.10	0.86	NA	
	52 weeks	RR: 1.10	0.80, 1.51	0.56	97%	
Clinically significant hypoglycaemia	26 weeks	RR: 0.99	0.93, 1.06	0.75	6%	Figure S43
	32 weeks	RR: 0.90	0.72, 1.12	0.36	NA	
	52 weeks	RR: 1.10	0.72, 1.68	0.66	92%	
Severe hypoglycaemia	26 weeks	RR: 1.89	0.38, 9.24	0.43	49%	Figure S44
	32 weeks	RR: 5.04	0.24, 103.94	0.3	NA	
	52 weeks	RR: 0.64	0.01, 29.26	0.82	85%	
Hypoglycaemia alert event rate	26 weeks	Rate ratio: 1.15	1.04, 1.27	0.006	0%	Figure S45
	32 weeks	Rate ratio: 0.74	0.55, 0.99	0.04	NA	
	52 weeks	Rate ratio: 1.17	1.06, 1.29	0.002	0%	
Clinically significant hypoglycaemia event rate	26 weeks	Rate ratio: 1.21	1.15, 1.27	< 0.00001	0%	Figure S46
	32 weeks	Rate ratio: 0.78	0.44, 1.39	0.4	NA	
	52 weeks	Rate ratio: 1.20	1.15, 1.25	< 0.00001	0%	
Severe hypoglycaemia event rate	26 weeks	Rate ratio: 3.23	1.42, 7.35	0.005	NA	Figure S47
	32 weeks	—	—	—	—	
	52 weeks	Rate ratio: 3.44	1.64, 7.22	0.001	NA	
Nocturnal hypoglycaemia alert	26 weeks	RR: 0.99	0.93, 1.05	0.67	0%	Figure S48
	32 weeks	RR: 0.84	0.73, 0.98	0.02	NA	
	52 weeks	RR: 1.08	0.99, 1.19	0.08	NA	
Nocturnal clinically significant hypoglycaemia	26 weeks	RR: 1.02	0.86, 1.21	0.81	0%	Figure S49
	32 weeks	RR: 0.98	0.77, 1.24	0.85	NA	
	52 weeks	—	—	—	—	
Nocturnal hypoglycaemia alert event rate	26 weeks	Rate ratio: 0.99	0.85, 1.15	0.91	85%	Figure S50
	32 weeks	Rate ratio: 0.57	0.41, 0.80	0.001	NA	
	52 weeks	Rate ratio: 1.04	0.85, 1.27	0.7	NA	
Nocturnal clinically significant hypoglycaemia event rate	26 weeks	Rate ratio: 0.94	0.67, 1.32	0.73	0%	Figure S51
	32 weeks	Rate ratio: 0.67	0.39, 1.14	0.14	NA	
	52 weeks	—	—	—	—	

##### 3.5.3.1 At 26 Weeks

Time below range (54–69 mg/dL) was significantly higher. Alert and clinically significant hypoglycaemia event rates were significantly increased. Severe hypoglycaemia event rate was markedly elevated.

##### 3.5.3.2 At 32 Weeks

Alert hypoglycaemia event rate was significantly reduced. Risk and event rate for alert nocturnal hypoglycaemia were significantly reduced.

##### 3.5.3.3 At 52 Weeks

Time below range (54–69 mg/dL) was significantly higher. Time above range (180–250 mg/dL) was significantly reduced. Alert and clinically significant hypoglycaemia event rates were significantly increased. Severe hypoglycaemia event rate was markedly elevated.

## 4 Discussion

This meta‐analysis, drawing primarily on CGM‐derived metrics, found that once‐weekly BIF provides glycaemic control broadly comparable to once‐daily insulin degludec across a range of core parameters. No significant differences were observed between the two insulins in within‐day or between‐day glycaemic variability, overall time in range, or most categories of time above and below range. Notably, BIF was associated with a modest but statistically significant increase in time spent in the 54–69 mg/dL range, alongside a borderline reduction in time spent in moderate hyperglycaemia (180–250 mg/dL). Meanwhile, assessments of hypoglycaemia frequency revealed no major differences in alert, clinically significant, or severe episodes; rate‐based analyses indicated a higher incidence of clinically significant and severe hypoglycaemia event rates with BIF. Subgroup analyses showed these trends were more pronounced among individuals with type 1 diabetes, those previously treated with insulin, and at longer follow‐up durations. These findings underscore that while BIF achieves similar overall glycaemic control to degludec, its safety profile raises concerns regarding hypoglycaemia, particularly in high‐risk populations, necessitating careful patient selection and ongoing monitoring.

BIF, a next‐generation once‐weekly basal insulin, addresses key limitations of daily insulin by offering prolonged action, reduced glycaemic variability and improved adherence. Its unique structure—featuring a single‐chain insulin fused to the Fc domain of IgG2—enhances stability and enables FcRn‐mediated recycling, allowing for sustained plasma levels and consistent glucose control [23]. The resulting flatter pharmacodynamic profile lowers the risk of hypoglycaemia while maintaining full insulin receptor activation. BIF also facilitates directly observed assisted therapy, offering a practical solution for individuals with visual, cognitive, or motor impairments—as well as for children and young adults—who may struggle with the burden of daily injections [24]. Once‐weekly basal insulin formulations, such as BIF, offer a practical solution to the challenges associated with daily insulin therapy by reducing the injection burden from 365 to just 52 per year [18]. This significant reduction alleviates both physical discomfort and psychological resistance, potentially improving adherence, facilitating earlier insulin initiation and enhancing self‐management and quality of life [25]. By decreasing injection frequency and minimising the adverse effects of repeated subcutaneous administration, BIF may help overcome clinical inertia and transform the management of diabetes [26].

Time in range (70–180 mg/dL) has emerged as a key indicator of glycaemic control, offering meaningful insight into daily glucose variability and its association with diabetes‐related complications [27]. Maintaining levels above 70% is widely recommended to reduce the risk of outcomes such as retinopathy and microalbuminuria [28]. In this analysis, BIF demonstrated comparable efficacy to insulin degludec in maintaining time in range, reinforcing its potential as a viable option, particularly for individuals facing challenges with adherence to daily insulin regimens. Additionally, BIF demonstrated comparable within‐day and between‐day glycaemic stability to insulin degludec. The flat pharmacokinetic and pharmacodynamic profile of BIF—achieved through structural modifications and Fc‐fusion technology—likely accounts for its comparable glycaemic stability to degludec. These design features enable sustained insulin release, reduced clearance, and minimal peak‐to‐trough fluctuation, thereby supporting stable glucose levels both within and between days [13, 29].

Hypoglycaemia remains a critical barrier to the effective use of insulin therapy, often deterring patients from initiating or adhering to treatment and posing therapeutic challenges for clinicians striving to balance glycaemic targets with safety [7]. In this analysis, no significant difference was observed between BIF and insulin degludec regarding the risk of alert, clinically significant, or nocturnal hypoglycaemia, a finding likely attributable to BIF's peakless pharmacodynamic profile that may alleviate concerns shared by both patients and clinicians [13]. However, BIF was associated with a significant increase in the event rates of clinically significant and severe hypoglycaemia, although nocturnal hypoglycaemia remained comparable across all evaluated subgroups. It is important to note that event rates appeared higher even when absolute risk differences were relatively modest, which could be due to repeated events occurring within individuals, thereby highlighting the distinction between the likelihood of experiencing an event and the frequency with which such events recur. These elevated risks were particularly evident among individuals with type 1 diabetes, potentially reflecting the limitations of once‐weekly formulations in accommodating the dynamic insulin requirements characteristic of this population [24]. Weekly basal insulin, while offering stable coverage, lacks the dosing flexibility of daily regimens, which may be crucial for individuals with highly variable insulin needs, especially those using advanced technologies such as closed‐loop systems. Such variability may underlie the increased susceptibility to hypoglycaemia observed in type 1 diabetes participants randomised to once‐weekly insulins across both studies [30]. This trend was also notable among insulin‐experienced individuals, who may have had less residual β‐cell function and more entrenched glycaemic patterns, further complicating the transition to a fixed weekly regimen. These findings underscore the importance of tailoring insulin therapy to individual patient profiles, as the fixed nature of weekly dosing may heighten the risk of hypoglycaemia in populations with complex or fluctuating insulin requirements, such as those with T1D or prior insulin use. However, in real‐world settings, these risks could be mitigated through careful dose titration, proactive monitoring and structured patient education [12, 31, 32]. Emphasising hypoglycaemia awareness, optimal timing of administration, and personalised adjustment strategies—along with clear support during the transition from daily to weekly regimens—may enhance the safe and effective implementation of BIF in clinical practice.

This meta‐analysis has several important limitations. Most included trials were not phase 3 and employed open‐label designs necessitated by differing insulin delivery devices and titration protocols, introducing potential performance bias. Heterogeneity was evident across studies due to variations in treatment duration, glycaemic targets and titration strategies. The pooling of insulin‐naive and insulin‐experienced individuals, as well as populations with both type 1 and type 2 diabetes, may have obscured treatment‐specific effects; however, this approach was necessitated by the limited number of available RCTs, which precluded separate meta‐analyses for these subgroups. The inclusion of only five RCTs, despite their methodological rigour, limits the generalisability of findings, particularly across diverse healthcare settings. Moreover, the absence of patient‐reported outcomes—such as treatment satisfaction, perceived convenience, or adherence to once‐weekly dosing—precludes insights into the lived experience of patients, which is critical for therapies involving novel delivery schedules. Finally, practical considerations—such as the reduced flexibility in dose adjustments, prolonged adverse effect duration, high cost, and limited availability—along with a lack of long‐term efficacy and safety data, constrain the immediate clinical applicability of once‐weekly basal insulin. Moreover, while formal cost comparisons were not reported, real‐world accessibility of BIF may be further shaped by manufacturing demands, regulatory approval processes, and pricing relative to established options like insulin degludec.

## 5 Conclusion

This meta‐analysis found that once‐weekly BIF provides glycaemic control comparable to daily insulin degludec but with increased mild hypoglycaemia (54–69 mg/dL) and higher event rates of clinically significant/severe hypoglycaemia—particularly in type 1 diabetes and insulin‐experienced patients. While BIF's weekly dosing may improve adherence, its hypoglycaemia risk necessitates cautious implementation with tailored dosing and monitoring.

## Author Contributions

All authors contributed meaningfully to this research through substantial involvement in study conception, design and execution. The team collectively conducted the systematic literature review, performed data extraction and quality assessment and carried out statistical analyses. Each member participated actively in interpreting results, drafting manuscript sections and providing critical revisions. All contributors reviewed and approved the final version of the paper, taking full responsibility for its content and integrity.

## Ethics Statement

The authors have nothing to report.

## Consent

The authors have nothing to report.

## Conflicts of Interest

The authors declare no conflicts of interest.

## Supporting information


**Figure S1.** Forest plot for within‐day glycaemic variability, comparing T1D and T2D subgroups.
**Figure S2.** Forest plot for between‐day glycaemic variability, comparing T1D and T2D subgroups.
**Figure S3.** Forest plot for time in range (70–180 mg/dL, %) in T1D and T2D subgroups.
**Figure S4.** Forest plot for time below range (< 54 mg/dL, %) in T1D and T2D subgroups.
**Figure S5.** Forest plot for time below range (54–69 mg/dL, %) in T1D and T2D subgroups.
**Figure S6.** Forest plot for time above range (180–250 mg/dL, %) in T1D and T2D subgroups.
**Figure S7.** Forest plot for time above range (> 250 mg/dL, %) in T1D and T2D subgroups.
**Figure S8.** Forest plot for alert hypoglycaemia in T1D and T2D subgroups.
**Figure S9.** Forest plot for clinically significant hypoglycaemia in T1D and T2D subgroups.
**Figure S10.** Forest plot for severe hypoglycaemia in T1D and T2D subgroups.
**Figure S11.** Forest plot for alert hypoglycaemia event rate in T1D and T2D subgroups.
**Figure S12.** Forest plot for clinically significant hypoglycaemia event rate in T1D and T2D subgroups.
**Figure S13.** Forest plot for severe hypoglycaemia event rate in T1D and T2D subgroups.
**Figure S14.** Forest plot for nocturnal alert hypoglycaemia in T1D and T2D subgroups.
**Figure S15.** Forest plot for nocturnal clinically significant hypoglycaemia in T1D and T2D subgroups.
**Figure S16.** Forest plot for nocturnal alert hypoglycaemia event rate in T1D and T2D subgroups.
**Figure S17.** Forest plot for nocturnal clinically significant hypoglycaemia event rate in T1D and T2D subgroups.
**Figure S18.** Forest plot for within‐day glycaemic variability (CV, %) comparing insulin‐naive and previously insulin‐treated participants.
**Figure S19.** Forest plot for between‐day glycaemic variability (CV, %) comparing insulin‐naive and previously insulin‐treated participants.
**Figure S20.** Forest plot for time in range (70–180 mg/dL, %) comparing insulin‐naive and previously insulin‐treated participants.
**Figure S21.** Forest plot for time below range (< 54 mg/dL, %) comparing insulin‐naive and previously insulin‐treated participants.
**Figure S22.** Forest plot for time below range (54–69 mg/dL, %) comparing insulin‐naive and previously insulin‐treated participants.
**Figure S23.** Forest plot for time above range (180–250 mg/dL, %) comparing insulin‐naive and previously insulin‐treated participants.
**Figure S24.** Forest plot for time above range (> 250 mg/dL, %) comparing insulin‐naive and previously insulin‐treated participants.
**Figure S25.** Forest plot for risk of hypoglycaemia alert comparing insulin‐naive and previously insulin‐treated participants.
**Figure S26.** Forest plot for risk of clinically significant hypoglycaemia comparing insulin‐naive and previously insulin‐treated participants.
**Figure S27.** Forest plot for risk of severe hypoglycaemia comparing insulin‐naive and previously insulin‐treated participants.
**Figure S28.** Forest plots for hypoglycaemia alert event rate comparing insulin‐naive and previously insulin‐treated participants.
**Figure S29.** Forest plot for clinically significant hypoglycaemia event rate comparing insulin‐naive and previously insulin‐treated participants.
**Figure S30.** Forest plot for severe hypoglycaemia event rate comparing insulin‐naive and previously insulin‐treated participants.
**Figure S31.** Forest plot for nocturnal hypoglycaemia alert comparing insulin‐naive and previously insulin‐treated participants.
**Figure S32.** Forest plot for nocturnal clinically significant hypoglycaemia comparing insulin‐naive and previously insulin‐treated participants.
**Figure S33.** Forest plot for nocturnal hypoglycaemia alert event rate comparing insulin‐naive and previously insulin‐treated participants.
**Figure S34.** Forest plot for nocturnal clinically significant hypoglycaemia event rate comparing insulin‐naive and previously insulin‐treated participants.
**Figure S35.** Forest plot for within‐day glycaemic variability (CV, %) at 26 and 52 weeks.
**Figure S36.** Forest plot for between‐day glycaemic variability (CV, %) at 26 and 52 weeks.
**Figure S37.** Forest plot for time in range (70–180 mg/dL, %) at 26, 32 and 52 weeks.
**Figure S38.** Forest plot for time below range (< 54 mg/dL, %) at 26 and 52 weeks.
**Figure S39.** Forest plot for time below range (54–69 mg/dL, %) at 26 and 52 weeks.
**Figure S40.** Forest plot for time above range (180–250 mg/dL, %) at 26 and 52 weeks.
**Figure S41.** Forest plot for time above range (> 250 mg/dL, %) at 26 and 52 weeks.
**Figure S42.** Forest plot for hypoglycaemia alert at 26, 32 and 52 weeks.
**Figure S43.** Forest plot for clinically significant hypoglycaemia at 26, 32 and 52 weeks.
**Figure S44.** Forest plot for severe hypoglycaemia at 26, 32 and 52 weeks.
**Figure S45.** Forest plot for hypoglycaemia alert event rate at 26, 32 and 52 weeks.
**Figure S46.** Forest plot for clinically significant hypoglycaemia event rate at 26, 32 and 52 weeks.
**Figure S47.** Forest plot for severe hypoglycaemia event rate at 26 and 52 weeks.
**Figure S48.** Forest plot for nocturnal hypoglycaemia alert at 26, 32 and 52 weeks.
**Figure S49.** Forest plot for nocturnal clinically significant hypoglycaemia at 26 and 32 weeks.
**Figure S50.** Forest plot for nocturnal hypoglycaemia alert event rate at 26, 32 and 52 weeks.
**Figure S51.** Forest plot for nocturnal clinically significant hypoglycaemia event rate at 26 and 32 weeks.

## Data Availability

All research data, including extracted datasets and analytical outputs, are fully available in the main document and Supporting Information.
