# Supplementary material for: Continuous Glucose Monitoring and Hypoglycaemia Metrics With Once‐Weekly Basal Insulin Fc Versus Insulin Degludec: A Systematic Review and Meta‐Analysis
Source: Endocrinol Diabetes Metab. 2025 Jun 13;8(4):e70067. doi: 10.1002/edm2.70067 (PMC12163248; doi:10.1002/edm2.70067)

**Supplementary Material**

**Supplementary Figure 1**: Forest plot for within-day glycemic variability, comparing T1D and T2D subgroups.

**Supplementary Figure 2**: Forest plot for between-day glycemic variability, comparing T1D and T2D subgroups.

**Supplementary Figure 3**: Forest plot for time in range (70–180 mg/dL, %) in T1D and T2D subgroups.

**Supplementary Figure 4**: Forest plot for time below range (<54 mg/dL, %) in T1D and T2D subgroups.

**Supplementary Figure 5**: Forest plot for time below range (54–69 mg/dL, %) in T1D and T2D subgroups.

**Supplementary Figure 6**: Forest plot for time above range (180–250 mg/dL, %) in T1D and T2D subgroups.

**Supplementary Figure 7**: Forest plot for time above range (>250 mg/dL, %) in T1D and T2D subgroups.

**Supplementary Figure 8**: Forest plot for alert hypoglycemia in T1D and T2D subgroups.

**Supplementary Figure 9**: Forest plot for clinically significant hypoglycemia in T1D and T2D subgroups.

**Supplementary Figure 10**: Forest plot for severe hypoglycemia in T1D and T2D subgroups.

**Supplementary Figure 11**: Forest plot for alert hypoglycemia event rate in T1D and T2D subgroups.

**Supplementary Figure 12**: Forest plot for clinically significant hypoglycemia event rate in T1D and T2D subgroups.

**Supplementary Figure 13**: Forest plot for severe hypoglycemia event rate in T1D and T2D subgroups.

**Supplementary Figure 14**: Forest plot for nocturnal alert hypoglycemia in T1D and T2D subgroups.

**Supplementary Figure 15**: Forest plot for nocturnal clinically significant hypoglycemia in T1D and T2D subgroups.

**Supplementary Figure 16**: Forest plot for nocturnal alert hypoglycemia event rate in T1D and T2D subgroups.

**Supplementary Figure 17**: Forest plot for nocturnal clinically significant hypoglycemia event rate in T1D and T2D subgroups.

**Supplementary Figure 18:** Forest plot for within-day glycemic variability (CV, %) comparing insulin-naive and previously insulin-treated participants.

**Supplementary Figure 19:** Forest plot for between-day glycemic variability (CV, %) comparing insulin-naive and previously insulin-treated participants.

**Supplementary Figure 20:** Forest plot for time in range (70–180 mg/dL, %) comparing insulin-naive and previously insulin-treated participants.

**Supplementary Figure 21:** Forest plot for time below range (<54 mg/dL, %) comparing insulin-naive and previously insulin-treated participants.

**Supplementary Figure 22:** Forest plot for time below range (54–69 mg/dL, %) comparing insulin-naive and previously insulin-treated participants.

**Supplementary Figure 23:** Forest plot for time above range (180–250 mg/dL, %) comparing insulin-naive and previously insulin-treated participants.

**Supplementary Figure 24:** Forest plot for time above range (>250 mg/dL, %) comparing insulin-naive and previously insulin-treated participants.

**Supplementary Figure 25:** Forest plot for risk of hypoglycemia alert comparing insulin-naive and previously insulin-treated participants.

**Supplementary Figure 26:** Forest plot for risk of clinically significant hypoglycemia comparing insulin-naive and previously insulin-treated participants.

**Supplementary Figure 27:** Forest plot for risk of severe hypoglycemia comparing insulin-naive and previously insulin-treated participants.

**Supplementary Figure 28:** Forest plots for hypoglycemia alert event rate comparing insulin-naive and previously insulin-treated participants.

**Supplementary Figure 29:** Forest plot for clinically significant hypoglycemia event rate comparing insulin-naive and previously insulin-treated participants.

**Supplementary Figure 30:** Forest plot for severe hypoglycemia event rate comparing insulin-naive and previously insulin-treated participants.

**Supplementary Figure 31:** Forest plot for nocturnal hypoglycemia alert comparing insulin-naive and previously insulin-treated participants.

**Supplementary Figure 32:** Forest plot for nocturnal clinically significant hypoglycemia comparing insulin-naive and previously insulin-treated participants.

**Supplementary Figure 33:** Forest plot for nocturnal hypoglycemia alert event rate comparing insulin-naive and previously insulin-treated participants.

**Supplementary Figure 34:** Forest plot for nocturnal clinically significant hypoglycemia event rate comparing insulin-naive and previously insulin-treated participants.

**Supplementary Figure 35.** Forest plot for within-day glycemic variability (CV, %) at 26 and 52 weeks.

**Supplementary Figure 36.** Forest plot for between-day glycemic variability (CV, %) at 26 and 52 weeks.

**Supplementary Figure 37.** Forest plot for time in range (70–180 mg/dL, %) at 26, 32, and 52 weeks.

**Supplementary Figure 38.** Forest plot for time below range (<54 mg/dL, %) at 26 and 52 weeks.

**Supplementary Figure 39.** Forest plot for time below range (54–69 mg/dL, %) at 26 and 52 weeks.

**Supplementary Figure 40.** Forest plot for time above range (180–250 mg/dL, %) at 26 and 52 weeks.

**Supplementary Figure 41.** Forest plot for time above range (>250 mg/dL, %) at 26 and 52 weeks.

**Supplementary Figure 42.** Forest plot for hypoglycemia alert at 26, 32, and 52 weeks.

**Supplementary Figure 43.** Forest plot for clinically significant hypoglycemia at 26, 32, and 52 weeks.

**Supplementary Figure 44.** Forest plot for severe hypoglycemia at 26, 32, and 52 weeks.

**Supplementary Figure 45.** Forest plot for hypoglycemia alert event rate at 26, 32, and 52 weeks.

**Supplementary Figure 46.** Forest plot for clinically significant hypoglycemia event rate at 26, 32, and 52 weeks.

**Supplementary Figure 47.** Forest plot for severe hypoglycemia event rate at 26 and 52 weeks.

**Supplementary Figure 48.** Forest plot for nocturnal hypoglycemia alert at 26, 32, and 52 weeks.

**Supplementary Figure 49.** Forest plot for nocturnal clinically significant hypoglycemia at 26 and 32 weeks.

**Supplementary Figure 50.** Forest plot for nocturnal hypoglycemia alert event rate at 26, 32, and 52 weeks.

**Supplementary Figure 51.** Forest plot for nocturnal clinically significant hypoglycemia event rate at 26 and 32 weeks.

**Supplementary Figure 1**: Forest plot for within-day glycemic variability, comparing T1D and T2D subgroups.


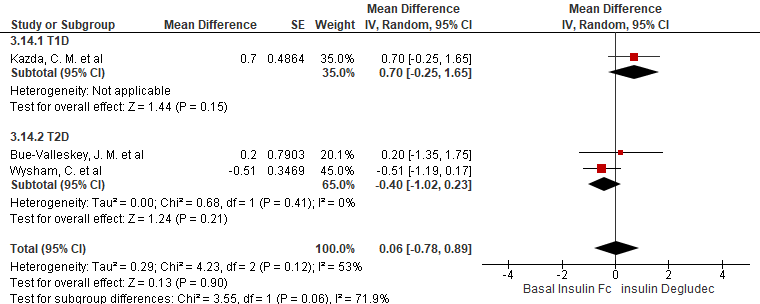


**Supplementary Figure 2**: Forest plot for between-day glycemic variability, comparing T1D and T2D subgroups.


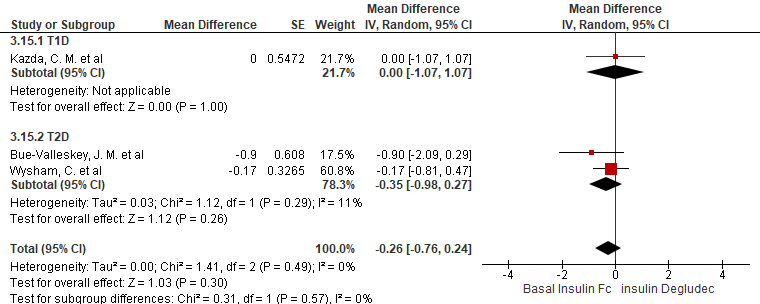


**Supplementary Figure 3**: Forest plot for time in range (70–180 mg/dL, %) in T1D and T2D subgroups.


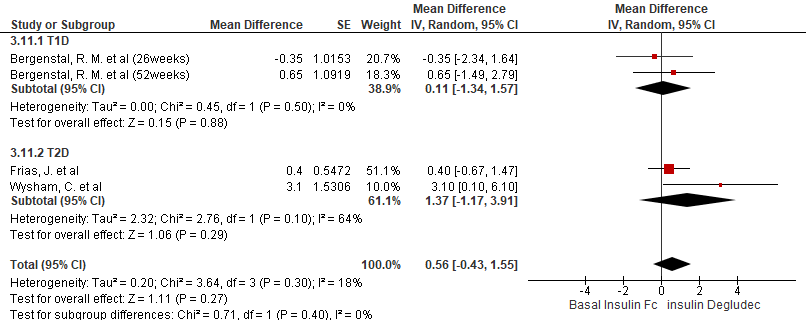


**Supplementary Figure 4**: Forest plot for time below range (<54 mg/dL, %) in T1D and T2D subgroups.


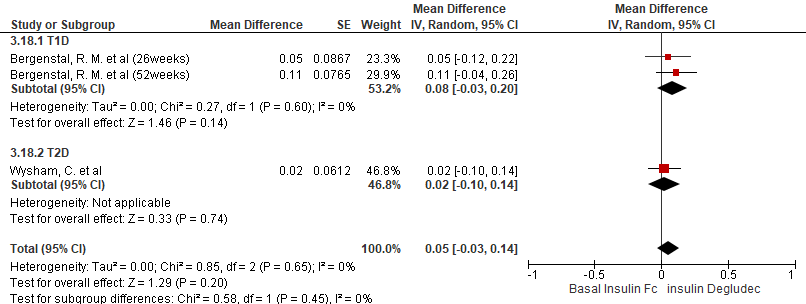


**Supplementary Figure 5**: Forest plot for time below range (54–69 mg/dL, %) in T1D and T2D subgroups.


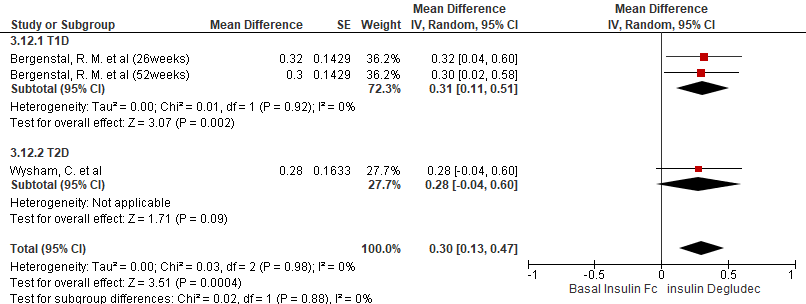


**Supplementary Figure 6**: Forest plot for time above range (180–250 mg/dL, %) in T1D and T2D subgroups.


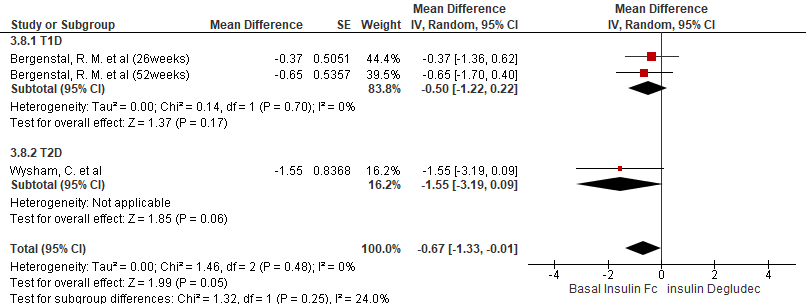


**Supplementary Figure 7**: Forest plot for time above range (>250 mg/dL, %) in T1D and T2D subgroups.


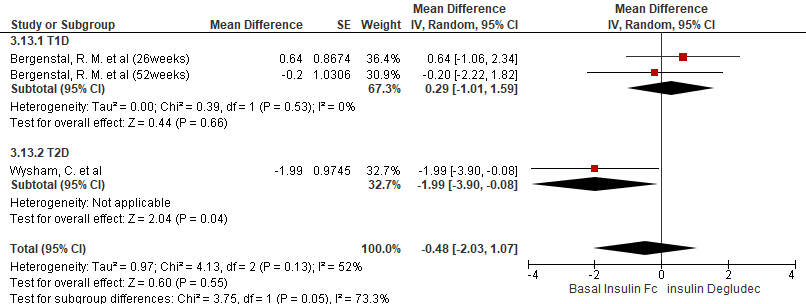


**Supplementary Figure 8**: Forest plot for hypoglycemia alert in T1D and T2D subgroups.


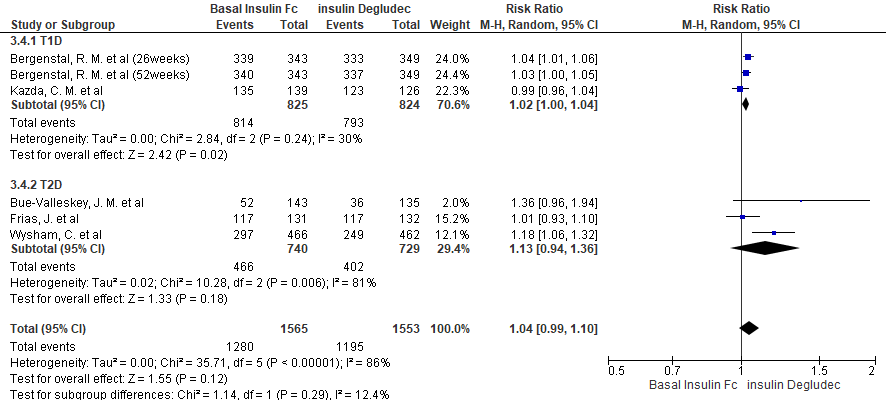


**Supplementary Figure 9**: Forest plot for clinically significant hypoglycemia in T1D and T2D subgroups.


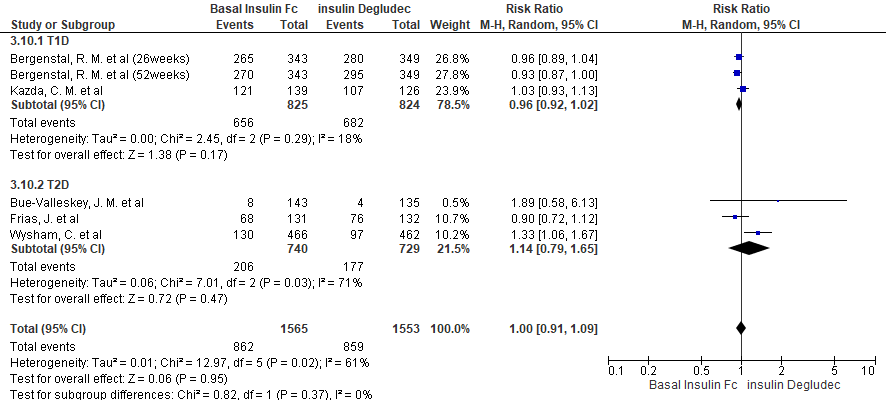


**Supplementary Figure 10**: Forest plot for severe hypoglycemia in T1D and T2D subgroups.


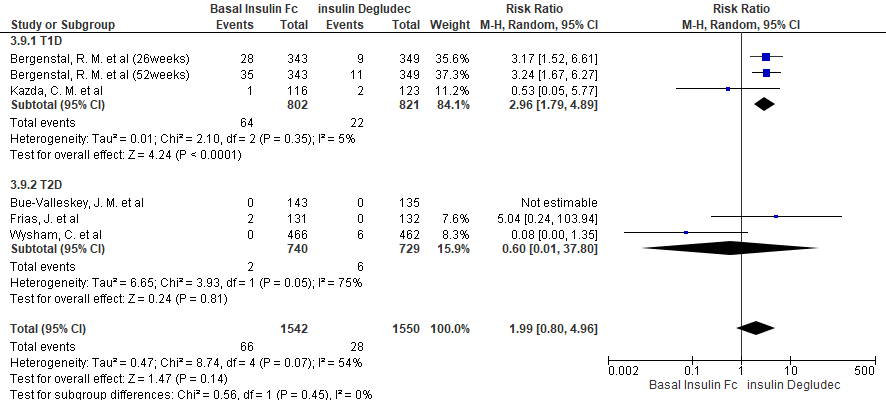


**Supplementary Figure 11**: Forest plot for hypoglycemia alert event rate in T1D and T2D subgroups.


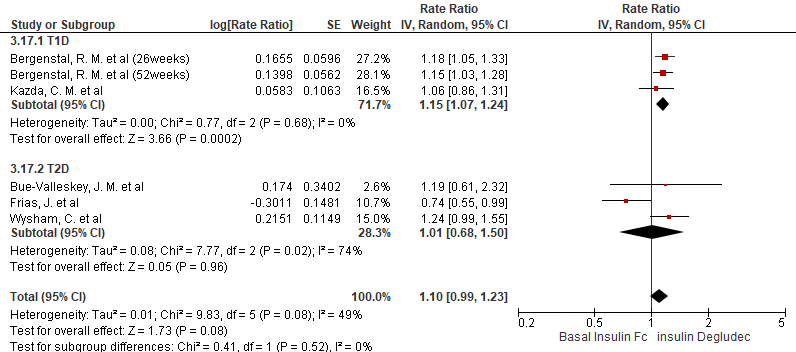


**Supplementary Figure 12**: Forest plot for clinically significant hypoglycemia event rate in T1D and T2D subgroups.


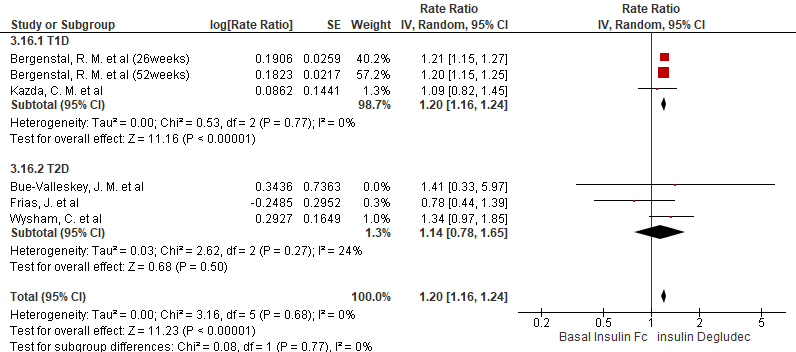


**Supplementary Figure 13**: Forest plot for severe hypoglycemia event rate in T1D subgroup.


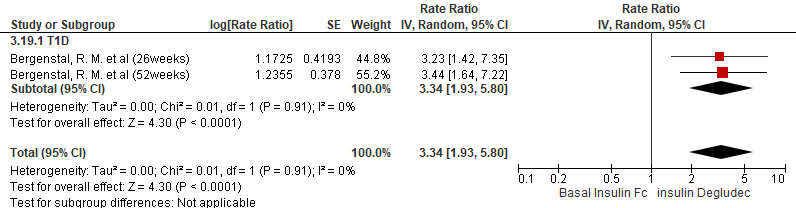


**Supplementary Figure 14**: Forest plot for nocturnal alert hypoglycemia in T1D and T2D subgroups.


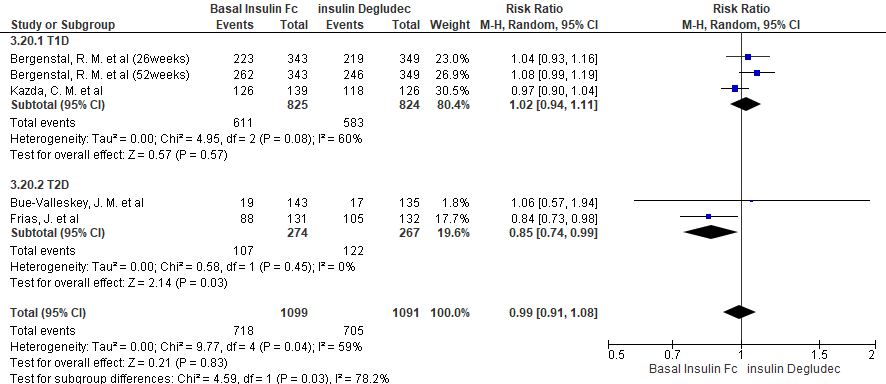


**Supplementary Figure 15**: Forest plot for nocturnal clinically significant hypoglycemia in T1D and T2D subgroups.


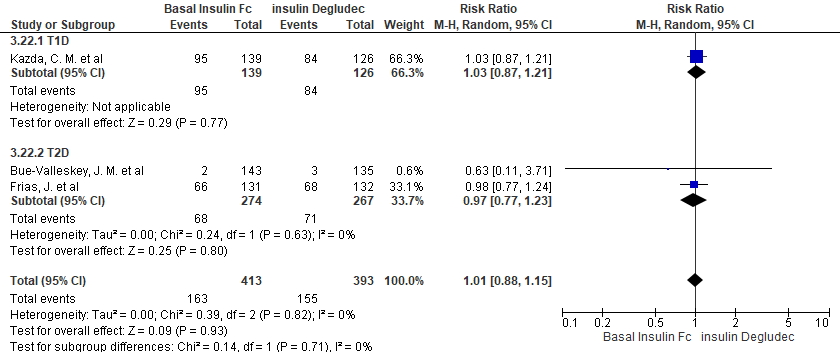


**Supplementary Figure 16**: Forest plot for nocturnal alert hypoglycemia event rate in T1D and T2D subgroups.


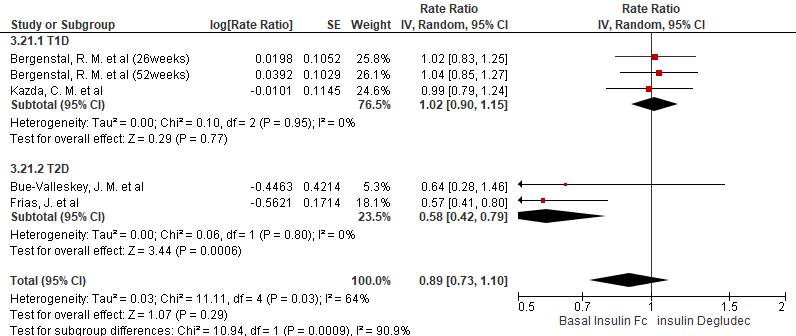


**Supplementary Figure 17**: Forest plot for nocturnal clinically significant hypoglycemia event rate in T1D and T2D subgroups.


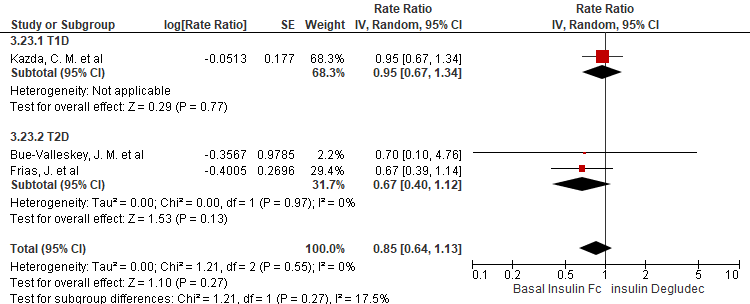


**Supplementary Figure 18:** Forest plot for within-day glycemic variability (CV, %) comparing insulin-naive and previously insulin-treated participants.


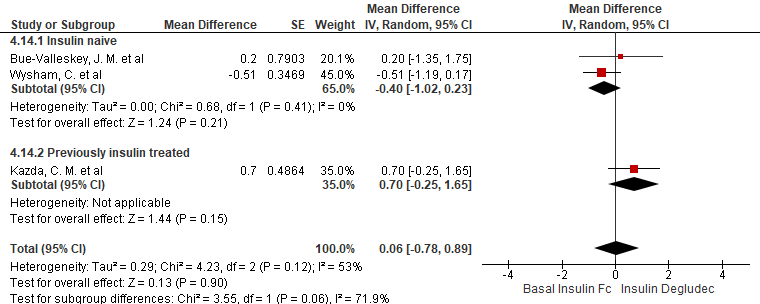


**Supplementary Figure 19:** Forest plot for between-day glycemic variability (CV, %) comparing insulin-naive and previously insulin-treated participants.


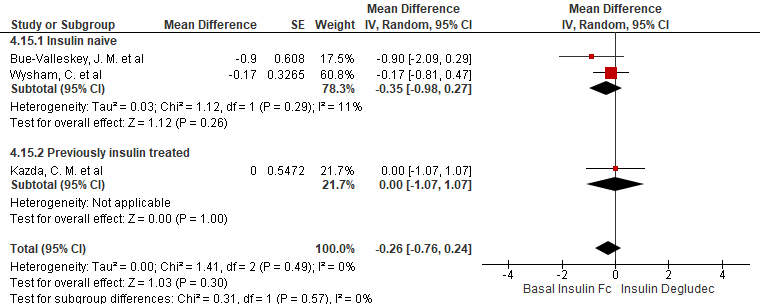


**Supplementary Figure 20:** Forest plot for time in range (70–180 mg/dL, %) comparing insulin-naive and previously insulin-treated participants.


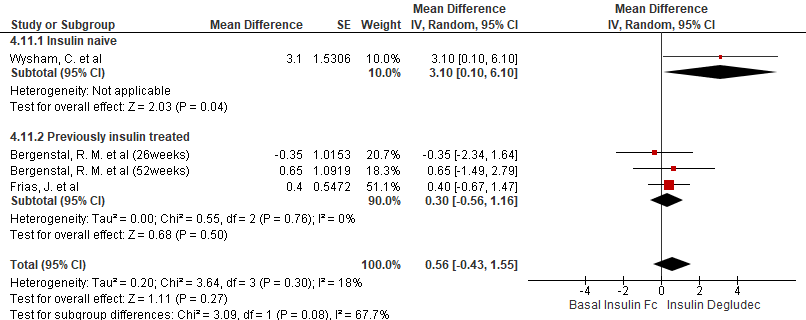


**Supplementary Figure 21:** Forest plot for time below range (<54 mg/dL, %) comparing insulin-naive and previously insulin-treated participants.


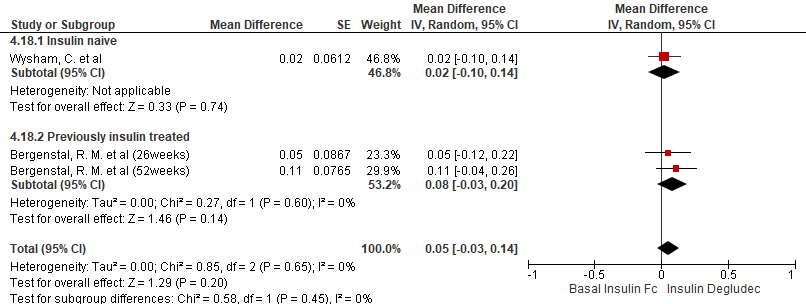


**Supplementary Figure 22:** Forest plot for time below range (54–69 mg/dL, %) comparing insulin-naive and previously insulin-treated participants.


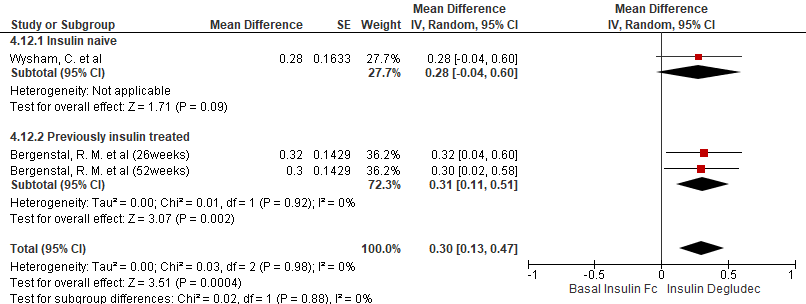


**Supplementary Figure 23:** Forest plot for time above range (180–250 mg/dL, %) comparing insulin-naive and previously insulin-treated participants.


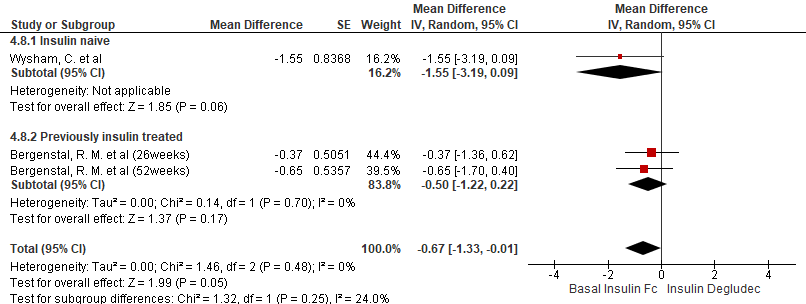


**Supplementary Figure 24:** Forest plot for time above range (>250 mg/dL, %) comparing insulin-naive and previously insulin-treated participants.


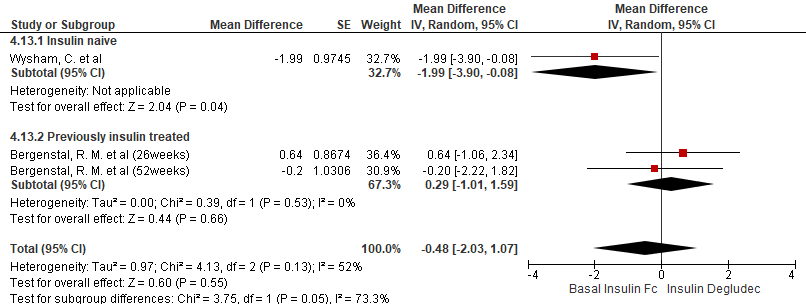


**Supplementary Figure 25:** Forest plot for risk of hypoglycemia alert comparing insulin-naive and previously insulin-treated participants.


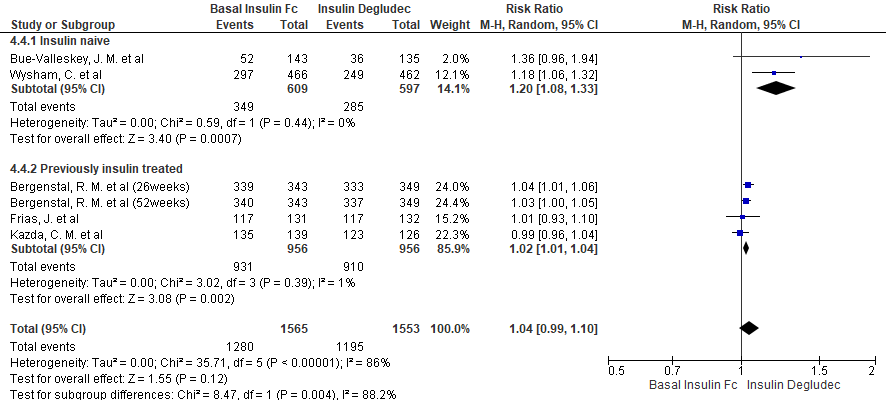


**Supplementary Figure 26:** Forest plot for risk of clinically significant hypoglycemia comparing insulin-naive and previously insulin-treated participants.


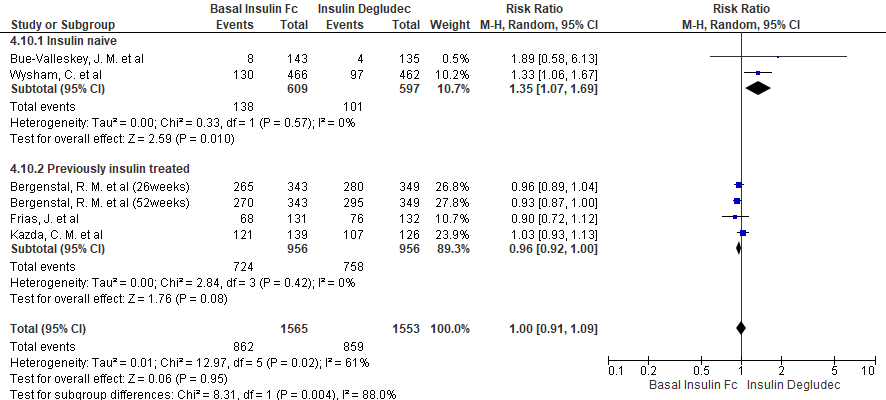


**Supplementary Figure 27:** Forest plot for risk of severe hypoglycemia comparing insulin-naive and previously insulin-treated participants.


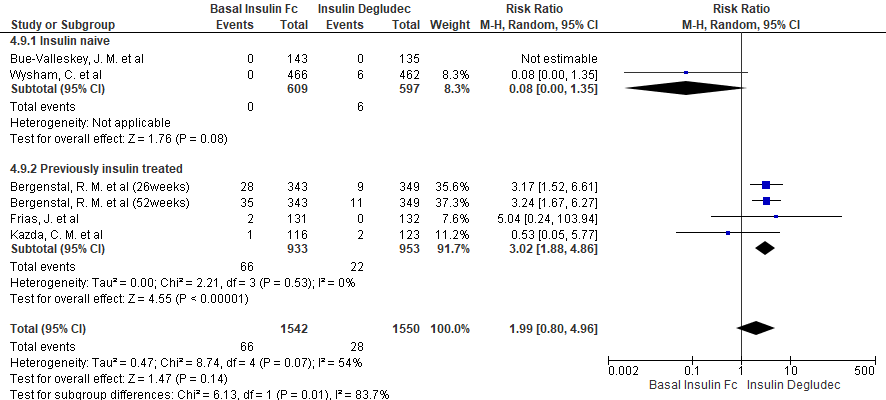


**Supplementary Figure 28:** Forest plots for hypoglycemia alert event rate comparing insulin-naive and previously insulin-treated participants.


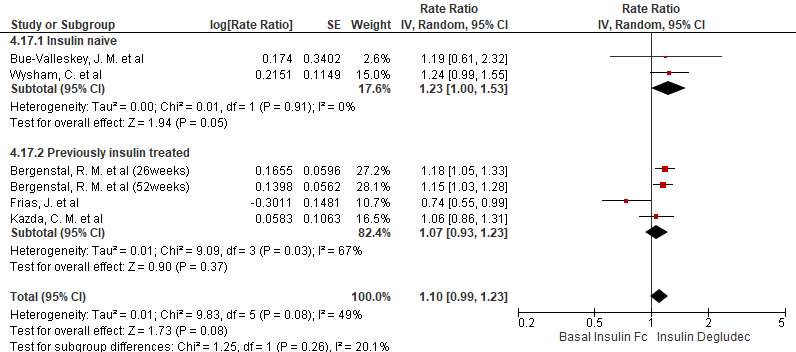


**Supplementary Figure 29:** Forest plots for clinically significant hypoglycemia event rate comparing insulin-naive and previously insulin-treated participants.


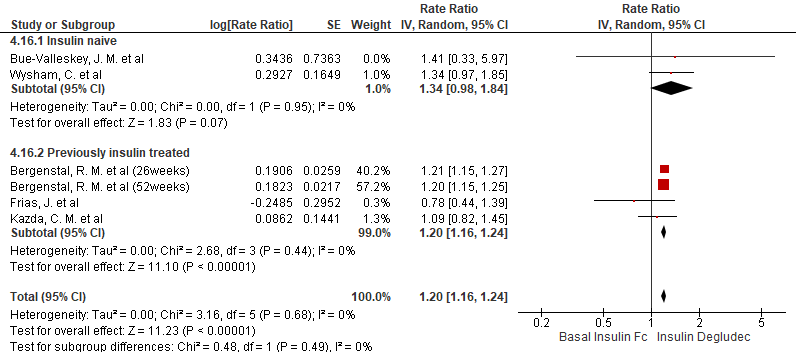


**Supplementary Figure 30:** Forest plot for severe hypoglycemia event rate in previously insulin-treated participants.


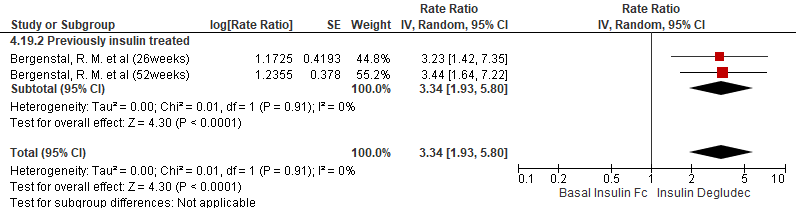


**Supplementary Figure 31:** Forest plot for nocturnal hypoglycemia alert comparing insulin-naive and previously insulin-treated participants.


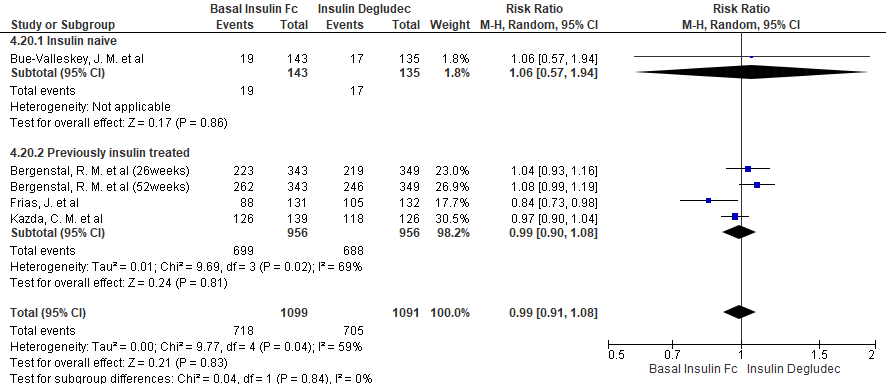


**Supplementary Figure 32:** Forest plot for nocturnal clinically significant hypoglycemia comparing insulin-naive and previously insulin-treated participants.


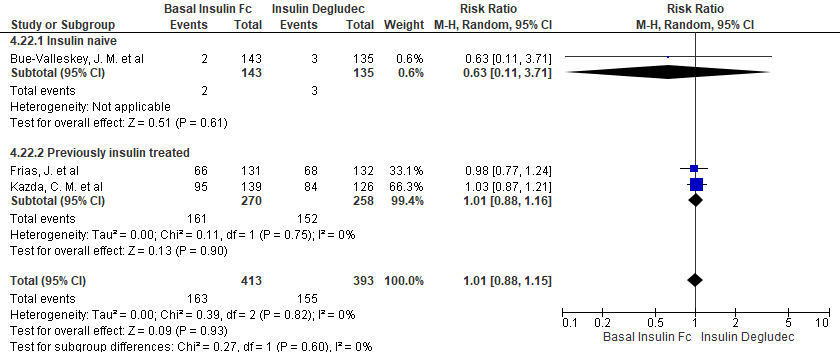


**Supplementary Figure 33:** Forest plot for nocturnal hypoglycemia alert event rate comparing insulin-naive and previously insulin-treated participants.


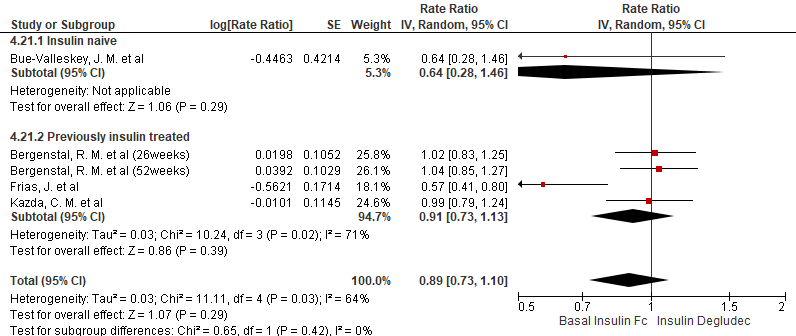


**Supplementary Figure 34:** Forest plot for nocturnal clinically significant hypoglycemia event rate comparing insulin-naive and previously insulin-treated participants.


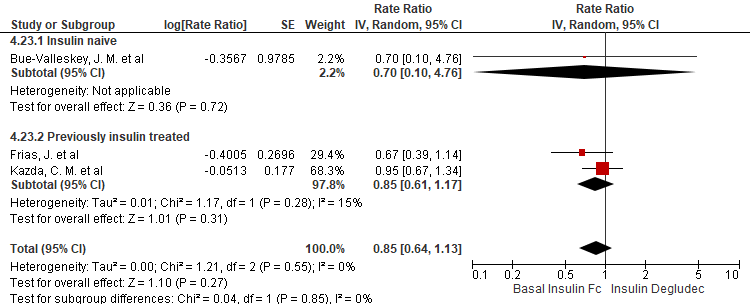


**Supplementary Figure 35.** Forest plot for within-day glycemic variability (CV, %) at 26 and 52 weeks.


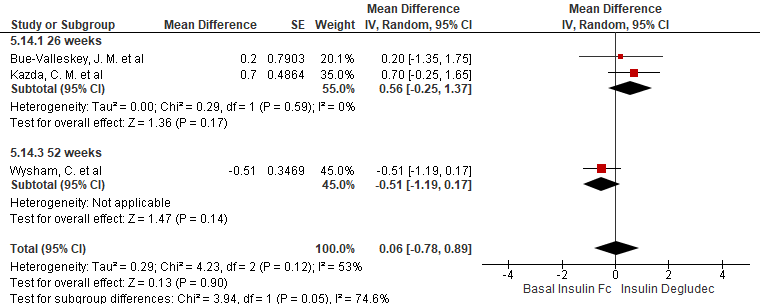


**Supplementary Figure 36.** Forest plot for between-day glycemic variability (CV, %) at 26 and 52 weeks.


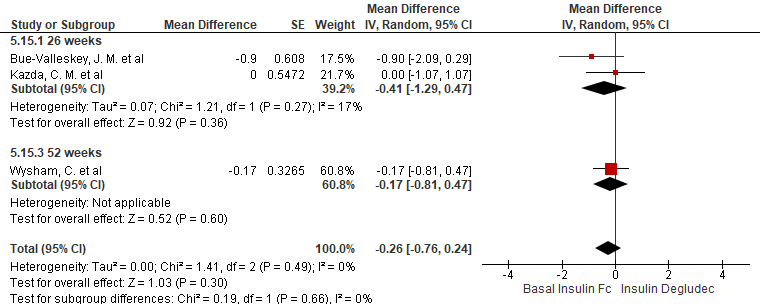


**Supplementary Figure 37.** Forest plot for time in range (70–180 mg/dL, %) at 26, 32, and 52 weeks.


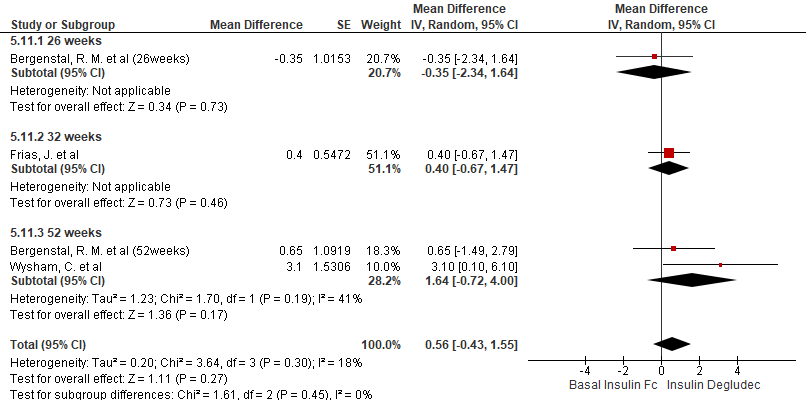


**Supplementary Figure 38.** Forest plot for time below range (<54 mg/dL, %) at 26 and 52 weeks.


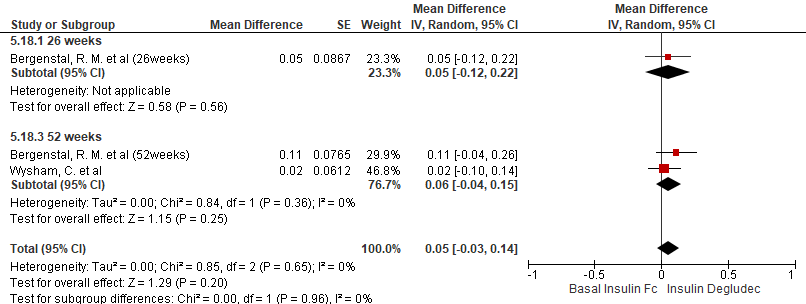


**Supplementary Figure 39.** Forest plot for time below range (54–69 mg/dL, %) at 26 and 52 weeks.


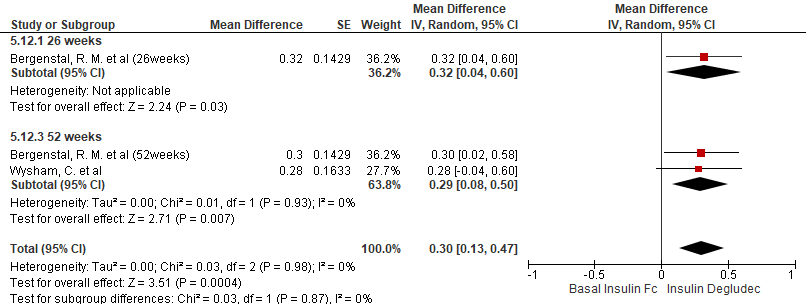


**Supplementary Figure 40.** Forest plot for time above range (180–250 mg/dL, %) at 26 and 52 weeks.


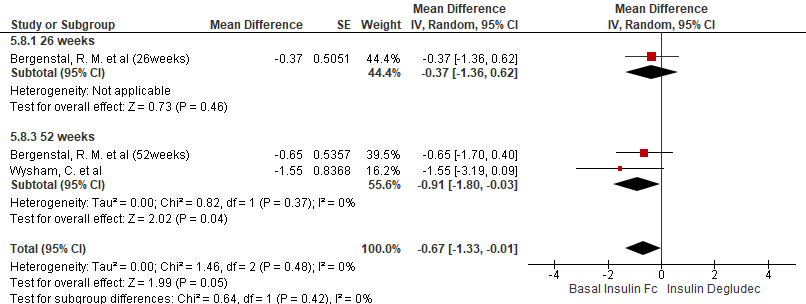


**Supplementary Figure 41.** Forest plot for time above range (>250 mg/dL, %) at 26 and 52 weeks.


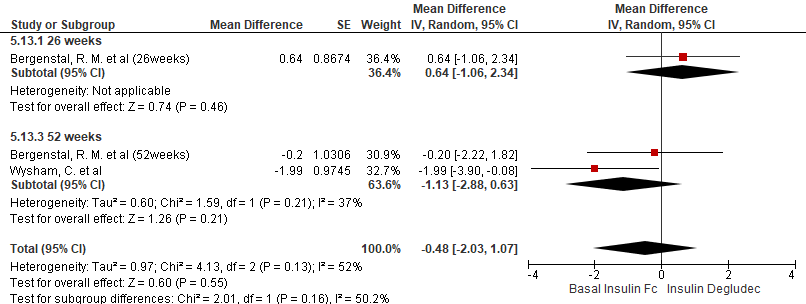


**Supplementary Figure 42.** Forest plot for hypoglycemia alert at 26, 32, and 52 weeks.


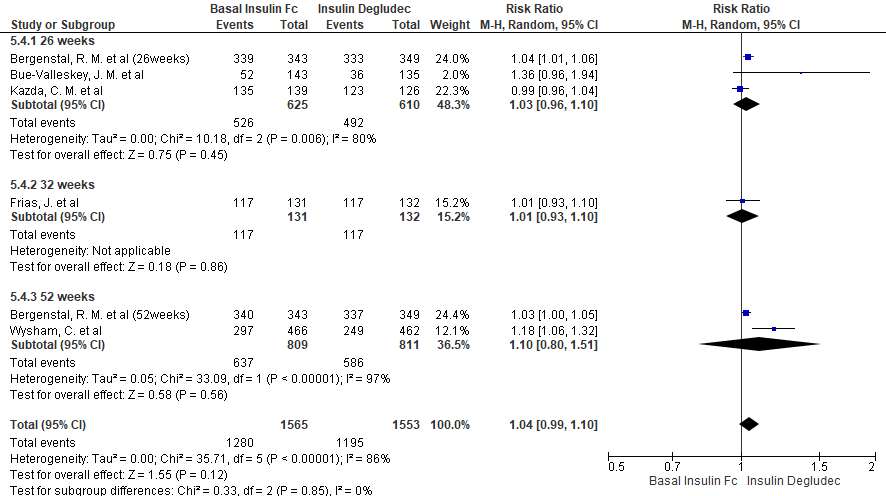


**Supplementary Figure 43.** Forest plot for clinically significant hypoglycemia at 26, 32, and 52 weeks.


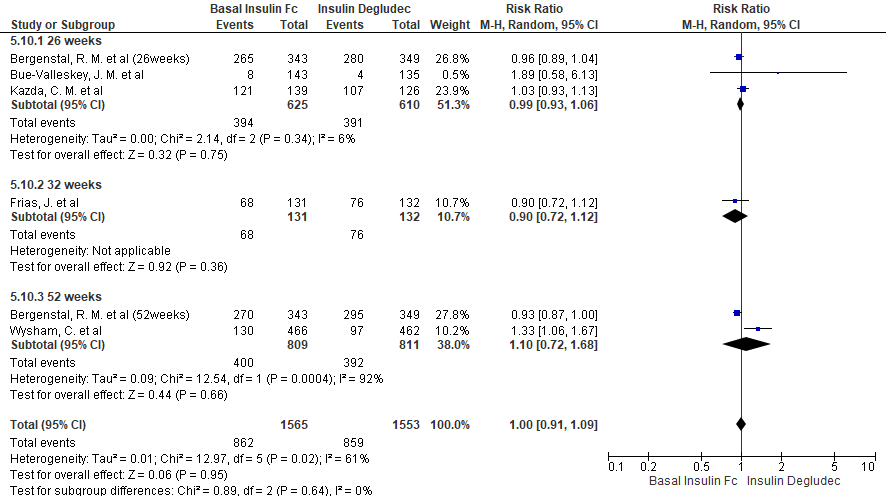


**Supplementary Figure 44.** Forest plot for severe hypoglycemia at 26, 32, and 52 weeks.


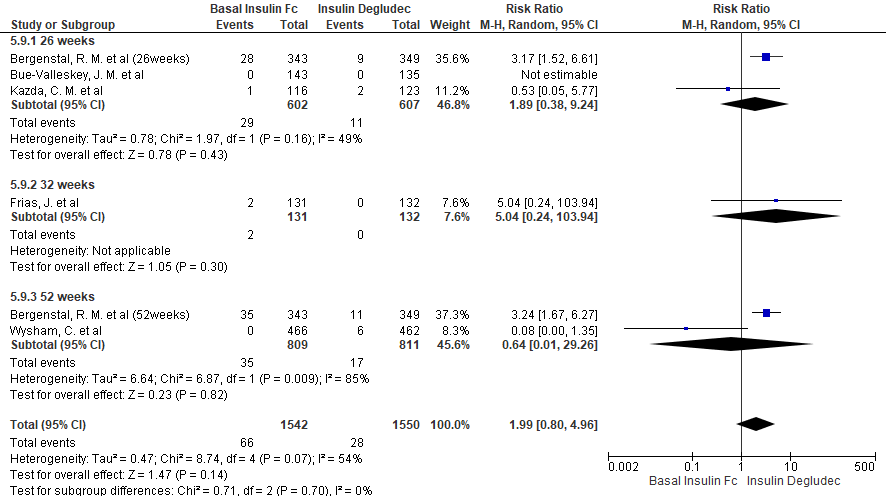


**Supplementary Figure 45.** Forest plot for hypoglycemia alert event rate at 26, 32, and 52 weeks.


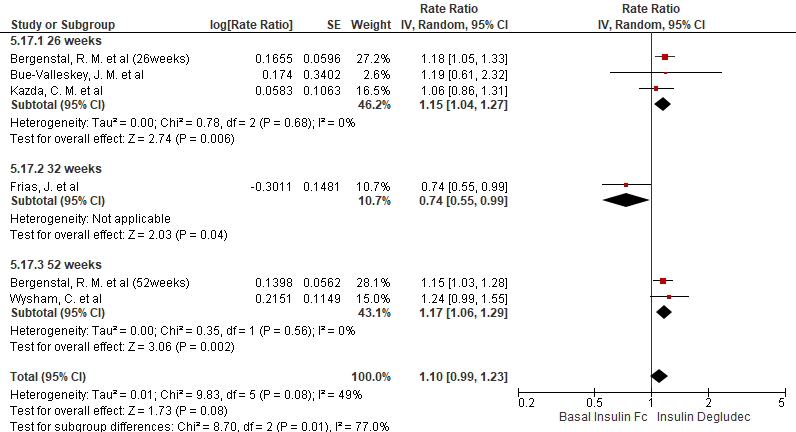


**Supplementary Figure 46.** Forest plot for clinically significant hypoglycemia event rate at 26, 32, and 52 weeks.


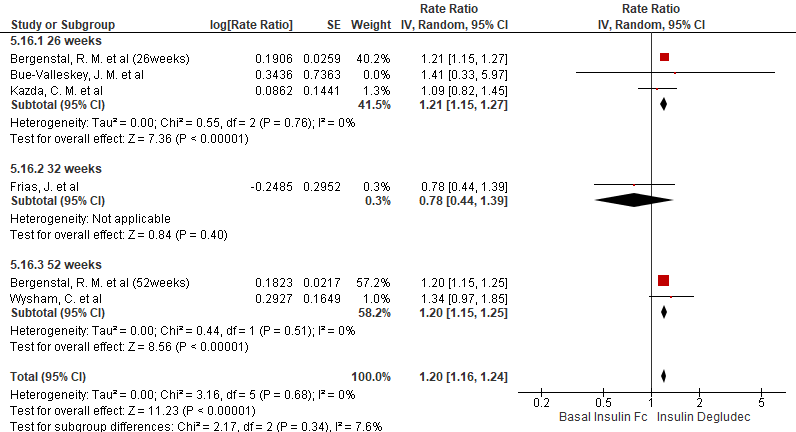


**Supplementary Figure 47.** Forest plot for severe hypoglycemia event rate at 26 and 52 weeks.


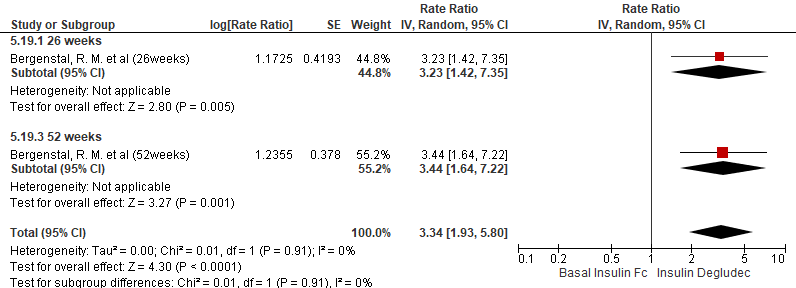


**Supplementary Figure 48.** Forest plot for nocturnal hypoglycemia alert at 26, 32, and 52 weeks.


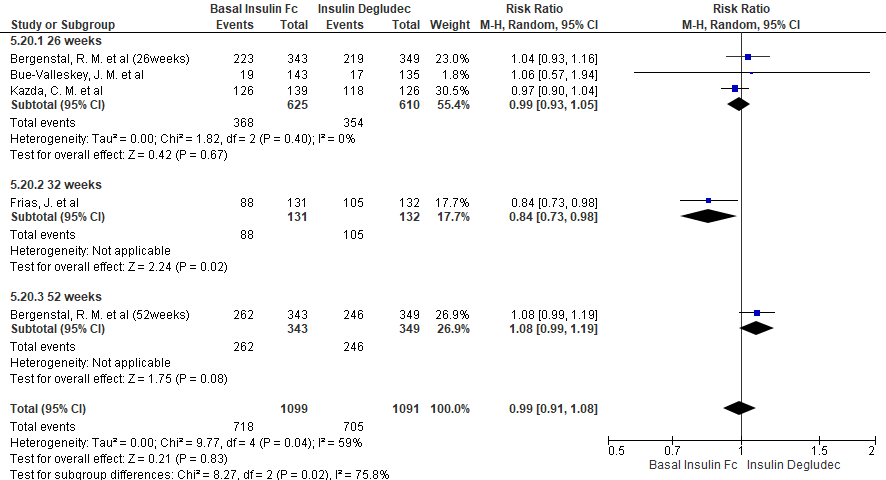


**Supplementary Figure 49.** Forest plot for nocturnal clinically significant hypoglycemia at 26 and 32 weeks.


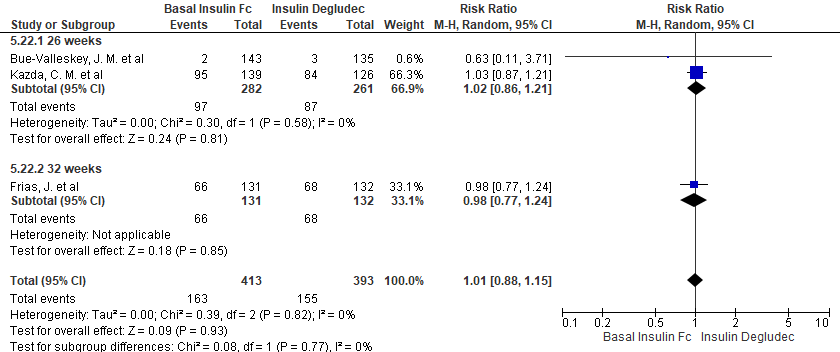


**Supplementary Figure 50.** Forest plot for nocturnal hypoglycemia alert event rate at 26, 32, and 52 weeks.


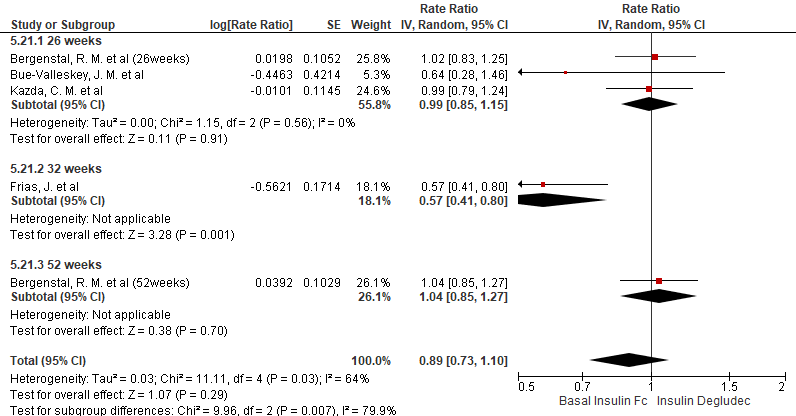


**Supplementary Figure 51.** Forest plot for nocturnal clinically significant hypoglycemia event rate at 26 and 32 weeks.


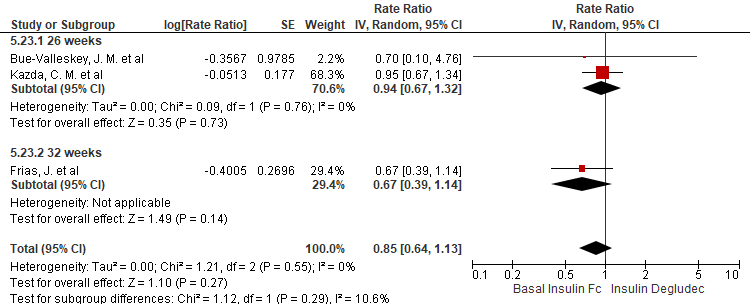

Supplement: Supplementary file 1 — Figure S1. Forest plot for within‐day glycaemic variability, comparing T1D and T2D subgroups. Figure S2. Forest plot for between‐day glycaemic variability, comparing T1D and T2D subgroups. Figure S3. Forest plot for time in range (70–180 mg/dL, %) in T1D and T2D subgroups. Figure S4. Forest plot for time below range (< 54 mg/dL, %) in T1D and T2D subgroups. Figure S5. Forest plot for time below range (54–69 mg/dL, %) in T1D and T2D subgroups. Figure S6. Forest plot for time above range (180–250 mg/dL, %) in T1D and T2D subgroups. Figure S7. Forest plot for time above range (> 250 mg/dL, %) in T1D and T2D subgroups. Figure S8. Forest plot for alert hypoglycaemia in T1D and T2D subgroups. Figure S9. Forest plot for clinically significant hypoglycaemia in T1D and T2D subgroups. Figure S10. Forest plot for severe hypoglycaemia in T1D and T2D subgroups. Figure S11. Forest plot for alert hypoglycaemia event rate in T1D and T2D subgroups. Figure S12. Forest plot for clinically significant hypoglycaemia event rate in T1D and T2D subgroups. Figure S13. Forest plot for severe hypoglycaemia event rate in T1D and T2D subgroups. Figure S14. Forest plot for nocturnal alert hypoglycaemia in T1D and T2D subgroups. Figure S15. Forest plot for nocturnal clinically significant hypoglycaemia in T1D and T2D subgroups. Figure S16. Forest plot for nocturnal alert hypoglycaemia event rate in T1D and T2D subgroups. Figure S17. Forest plot for nocturnal clinically significant hypoglycaemia event rate in T1D and T2D subgroups. Figure S18. Forest plot for within‐day glycaemic variability (CV, %) comparing insulin‐naive and previously insulin‐treated participants. Figure S19. Forest plot for between‐day glycaemic variability (CV, %) comparing insulin‐naive and previously insulin‐treated participants. Figure S20. Forest plot for time in range (70–180 mg/dL, %) comparing insulin‐naive and previously insulin‐treated participants. Figure S21. Forest plot for time below range (< 54 mg/dL, %) compari [file EDM2-8-e70067-s001.docx]
